# Supplementary material for: Identification of plasmon-driven nanoparticle-coalescence-dominated growth of gold nanoplates through nanopore sensing
Source: Nat Commun. 2022 Mar 17;13:1402. doi: 10.1038/s41467-022-29123-9 (PMC8931024; doi:10.1038/s41467-022-29123-9)
Supplement: Supplementary file 1 — Supplementary Information [file 41467_2022_29123_MOESM1_ESM.pdf]

Supplementary Information: Identification of Plasmon-driven Nanoparticle-coalescence-dominated Growth of Gold Nanoplates through Nanopore Sensing

Bintong Huang[1#], Longfei Miao[1#], Jing Li[1], Zhipeng Xie[1], Yong Wang[2], Jia Chai[1], and Yueming Zhai[1*]

[1]The Institute for Advanced Studies (IAS), Wuhan University, Wuhan, 430072, P. R. China.

[2]Shanghai Institute for Advanced Study, Institute of Quantitative Biology, College of Life Sciences, Zhejiang University, Hangzhou, 310027, P. R. China.

[#]These authors contributed equally.

*Corresponding author: yueming@whu.edu.cn

Supplementary Notes

Material: Hydrogen tetrachloroaurate(III) trihydrate ($HAuCl_4 \cdot 3H_2O$, ≥99.9%), iodine (I_2, 99.99%), potassium iodide (KI, 99%), sodium citrate ($Na_3C_6H_5O_7$, ≥98%), hexadecyltrimethylammonium chloride (CTAC, 99%), tetraethoxysilane (TEOS, 99%) were purchased from Adamas Reagent, Ltd (Shanghai, China). Sodium tetrahydroborate ($NaBH_4$), nitric acid (HNO_3), hydrochloric acid (HCl), sulfuric acid (H_2SO_4), sodium chloride (NaCl), potassium nitrate (KNO_3), hydrogen peroxide (H_2O_2), acetone, and ethanol triethanolamine (TEA) were of analytical grade and obtained from Sinopharm Chemical Reagent Co.,Ltd (Shanghai, China). All chemicals were used as received without further purification. Ultrapure water (18.2 MΩ cm) used in the experiments was provided by Standard arium® mini plus (Gottingen, Germany). All glassware was washed with *aqua regia* solution and rinsed thoroughly with ultrapure water prior to use. Caution! *Aqua regia* is highly corrosive and toxic: handle with care and use appropriate personal protection equipment.

Instrumentation: The Ultraviolet−visible (UV-Vis) spectra of samples were obtained on a spectrophotometer (LAMBDA-365, PerkinElmer). Field emission scanning electron microscope (SEM) was performed using Zeiss SIGMA, operating at 10 kV for Au samples and 5 kV for nanopipettes. Transmission electron microscopy (TEM) images were obtained using a JEM-F200 with an acceleration voltage of 200 kV. Structural and morphological characterization of the samples was performed on carbon-coated copper grids, and the transformation process was traced by a 30 nm thick SiNx TEM window (CleanSiN, YW MEMS). The light source used for the plasmon-driven conversion was a 300 W xenon lamp

(PLS-SXE300, Perfect light) equipped with different single-wavelength bandpass filters. The light intensity was measured by an optical power meter (PL-MW2000, Perfect light). Electrical measurements were performed using a patch clamp amplifier (Axopatch 200B, Molecular Device). The ionic current was converted to the digital signal by Axon Digidata 1550B (Molecular Device) and recorded by software Axon pCLAMP 10.4 (Molecular Device).

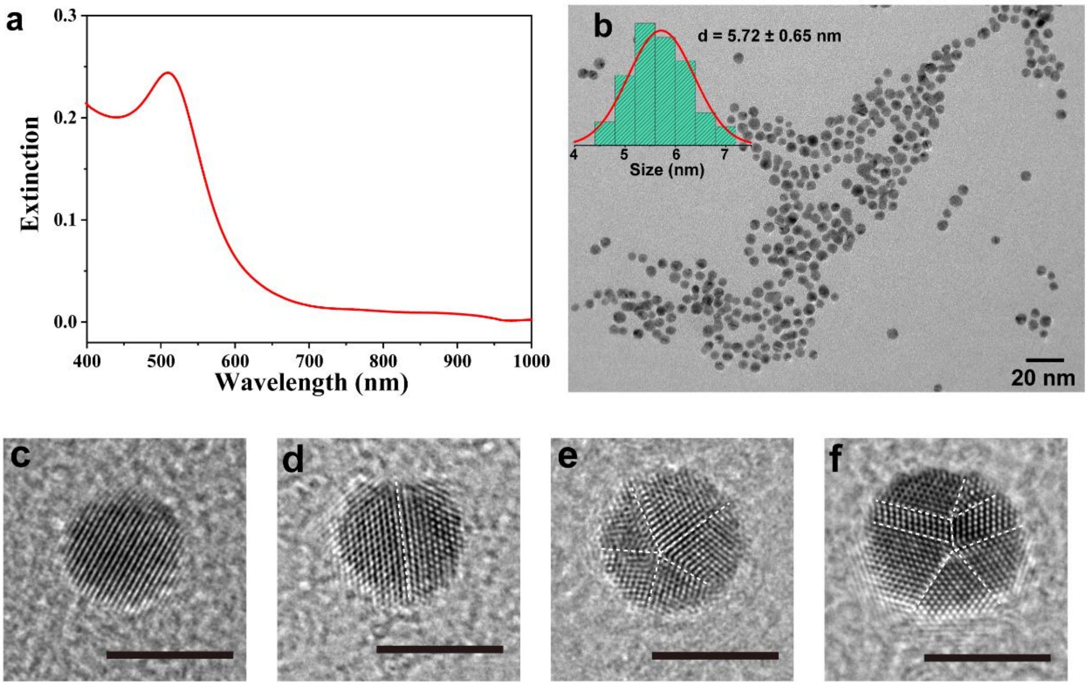

Supplementary Figure 1. Characterization of Au nanospheres. (a) UV–Vis spectrum of the Au nanospheres solution showing a prominent SPR feature at 512 nm. (b) TEM image of the as-prepared Au nanospheres. The inset histogram of Au nanoparticle sizes exhibits a rough Gaussian distribution with an average diameter of d=5.72 ± 0.65 nm. Data are presented as mean values ± s.d. (n = 100). HRTEM images of Au nanospheres: (c) single-crystalline (d) planar-twinned, (e) penta-twinned, and (f) multiply-twinned structure. The white dotted lines as a guide highlight the boundaries of the twins. All scale bars represent 5 nm in c-f.

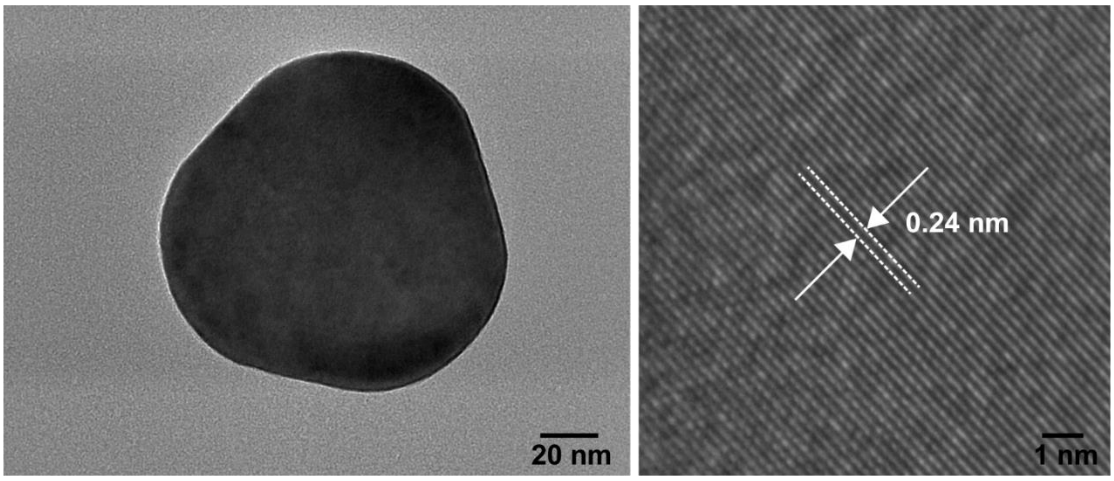

Supplementary Figure 2. Characterization of Au nanoplates. HRTEM image of the Au nanoplates displays a lattice spacing of 0.24 nm, in consistent with the {111} facet.

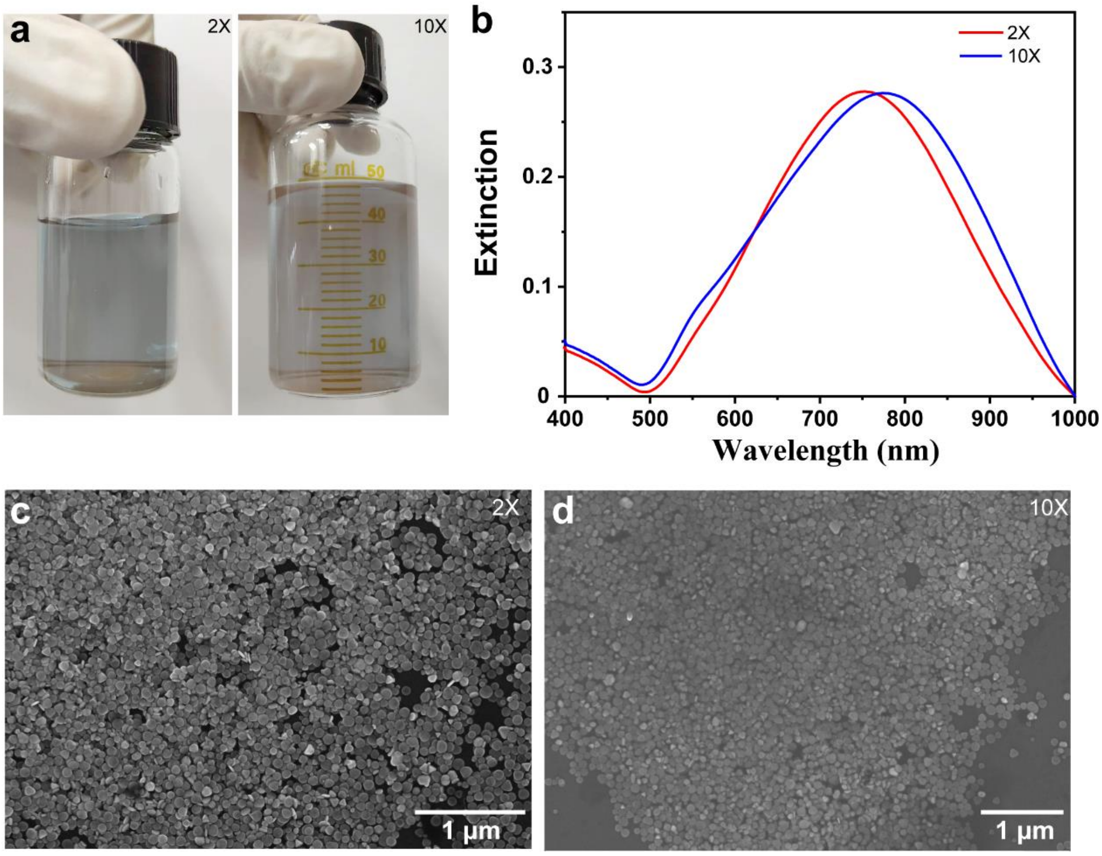

Supplementary Figure 3. Magnification experiments for the conversion of Au nanospheres to nanoplates. (a) Photograph of reaction solution (2X and 10X) under continuous light (500 ± 10 nm) irradiation 540 min. 2X reaction solution contains 10 ml of Au nanospheres solution and 340 μl of aqueous iodine (I_2, 0.5 mM). 10X reaction solution contains 50 ml of Au nanospheres solution and 1700 μl of aqueous iodine (I_2, 0.5 mM). (b) UV–Vis spectra of solution (2X and 10X). (c, d) SEM images of 2X and 10X. The high-yield Au nanoplates prepared by photochemical conversion process have good specificity and controllability in scale-up synthesis.

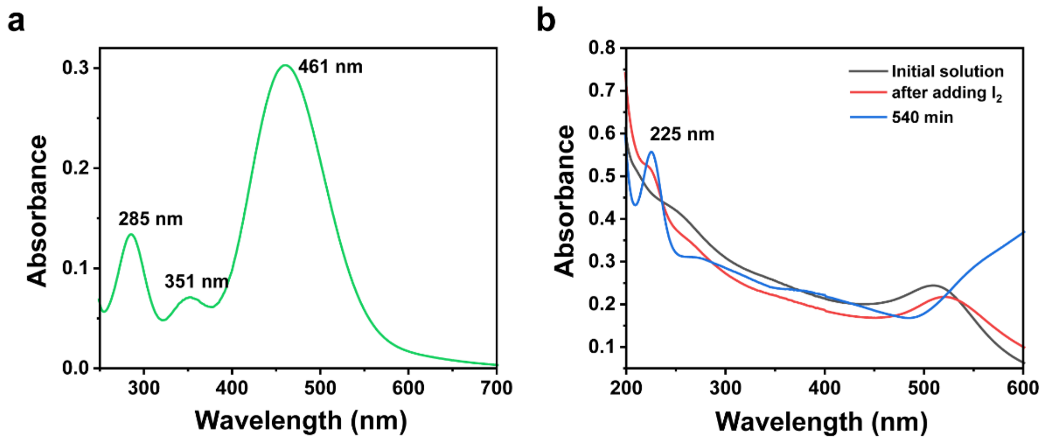

Supplementary Figure 4. Identification of species in aqueous iodine solutions and chemical oxidation etching process. (a) UV−vis spectrum for 0.5 mM iodine in aqueous solution at room temperature. (b) UV−vis spectra for the initial Au nanospheres solution, the reaction solution after adding 16.4 μM I_2, and light (500 ± 10 nm) irradiation for 540 min.

The following chemical reaction occurs when iodine dissolves in aqueous solution[1].

$I_2 + H_2O \rightleftharpoons HIO + H^+ + I^-$

$I_2 + 2\ OH^- \rightleftharpoons IO^- + H_2O + I^-$

$3\ IO^- \rightleftharpoons 2\ I^- + IO_3^-$

$I^- + I_2 \rightleftharpoons I_3^-$

Iodous acid and iodite are unstable and exist only as a transient intermediate. The only important polyiodide anion in aqueous solution is linear triiodide, I_3^-. As shown in **a**, the peaks at 285 nm and 351 nm correspond to I_3^- species and 461 nm to molecular iodine[2,3]. When I_2 aqueous solution was added to Au nanospheres solution, a new peak appeared at 225 nm corresponding to I^-. Therefore, we assume that Au^0 undergoes a very rapid chemical oxidation etching process:

$2Au^0 + I_3^- \rightleftharpoons 2AuI + I^-$

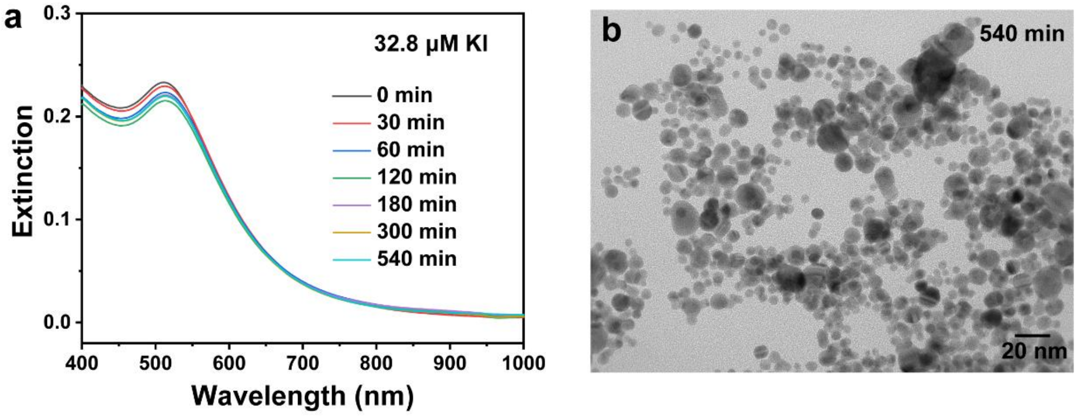

Supplementary Figure 5. Effect of iodide (KI) on conversion of Au nanospheres to nanoplates. (a) Time-dependent UV−vis spectra of Au nanoparticles obtained in the presence of 32.8 μM of KI. (b) TEM images of gold nanostructures from the reaction solution by illumination for 540 min. After 540 min of reaction, the SPR peak of the Au nanoplates was not observed. The TEM image also reveals that no Au nanoplates are found in the 540 min product, only some irregularly aggregated nanoparticles. I^- could first be oxidized to I_2 by an oxidant, such as dissolved oxygen[4,5].

$4I^- + O_2 + 2H_2O \rightleftharpoons 2I_2 + 4OH^-$

$I_2 + I^- \rightleftharpoons I_3^-$

$2Au^0 + I_3^- + I^- \rightleftharpoons 2AuI_2^-$

As the reaction progresses, dissolved oxygen is consumed and the etching ability is weakened. As a result, 32.8 μM of KI only leads to irregular aggregation of particles and no high yield of Au nanoplates are generated.

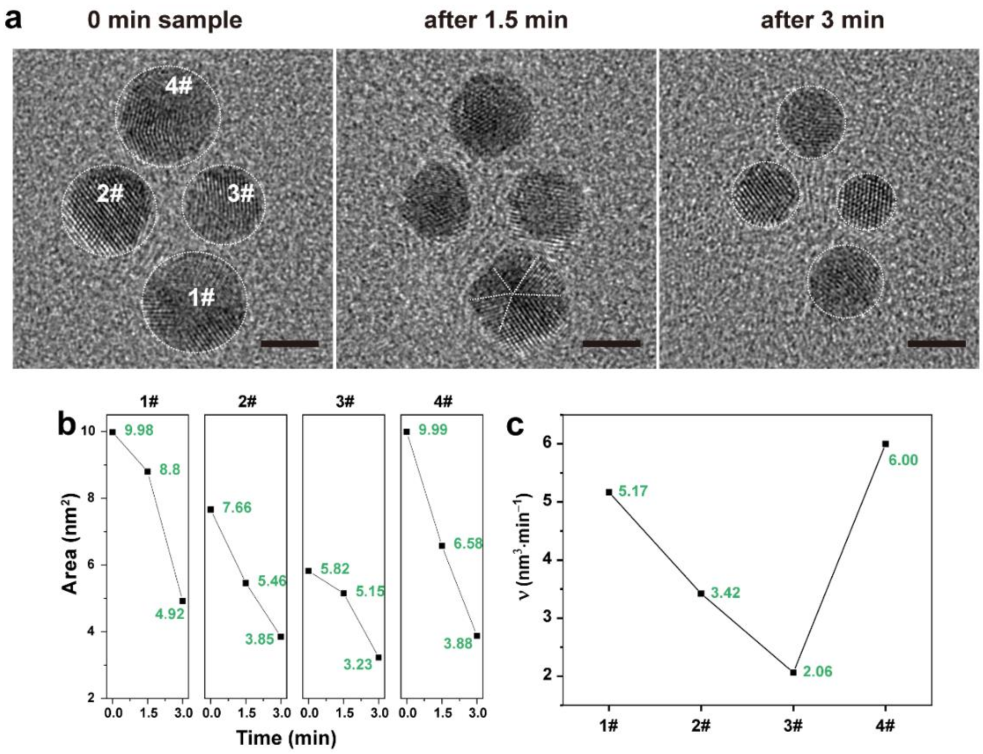

Supplementary Figure 6. Comparison of I_2 etching rates for different nanocrystal structures. The etching rates of different nanocrystal structures have been directly observed by TEM tracking Au nanospheres immobilized on SiNx window. (a) TEM images of Au nanospheres treated with I_2 for different times. All scale bars represent 2 nm. (b) The areas of the Au nanoparticles are calculated by assuming a spherical shape for the nanoparticles. (c) The etching rate was calculated:

$$\nu_{etching} = \frac{\frac{4}{3}\pi\left({R_0}^3 - {R_t}^3\right)}{t} = \frac{4}{3t\pi^{\frac{1}{2}}}\left(A_0^{\frac{3}{2}} - A_t^{\frac{3}{2}}\right)$$

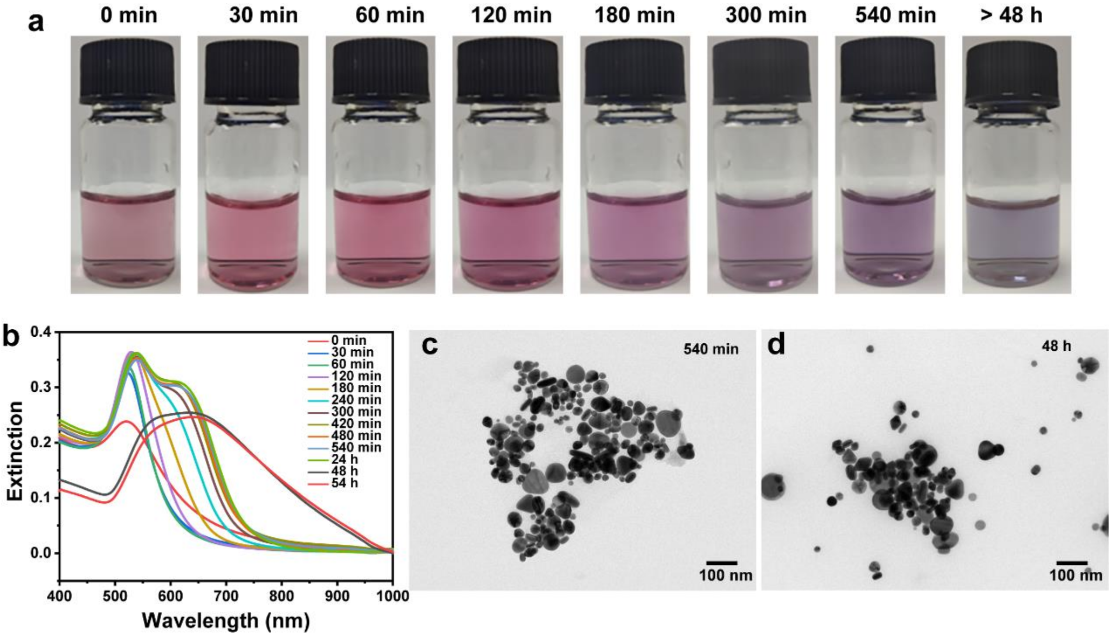

Supplementary Figure 7. Conversion Au nanospheres to nanoplates in the dark. (a) Photograph of the initial Au nanospheres solution, and the reaction solution in the dark after adding 16.4 μM I_2. (b) Time-dependent UV−vis spectra of conversion solution. (c-d) TEM images of Au nanostructures from the reaction solution in the dark at 540 min and 48 h. The color of the solution gradually changed from orange-red to purple, but it did not change to blue-green as happened under light irradiation condition. No high yield of Au nanoplates was generated in the final products (540 min and 48 h). Also, the characteristic SPR peak of Au nanoparticles did not disappear. The above results all indicate that light irradiation plays an important role in the shape conversion process.

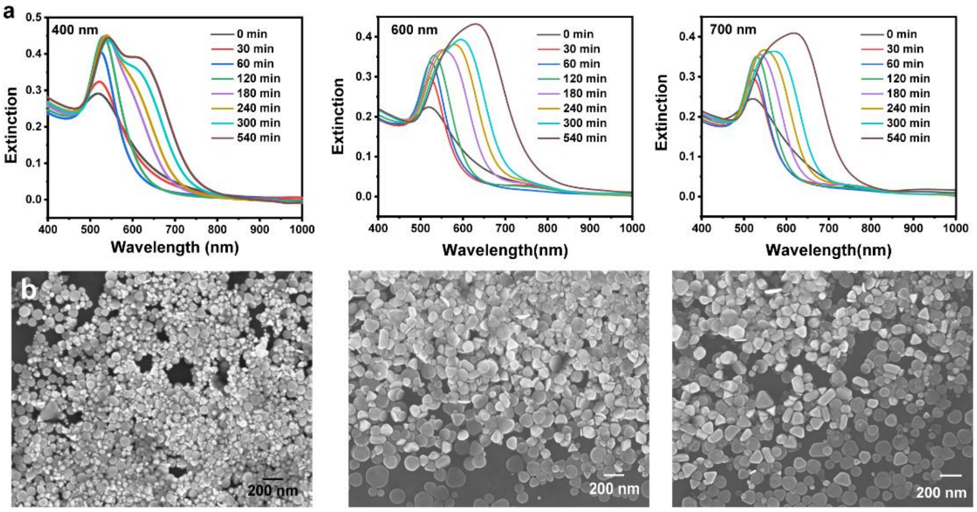

Supplementary Figure 8. Influence of incident excitation wavelength on the conversion of Au nanospheres to nanoplates. (a) Time-dependent UV-Vis spectra of reaction solution with 400 ± 10 nm, 600 ± 10 nm and 700 ±10 nm incident wavelengths of light. (b) SEM images of the products after 9 hours of irradiation with the corresponding incident wavelength of light. SEM images exhibited the formation of disk-like nanoplates and irregular shapes of nanoparticles.

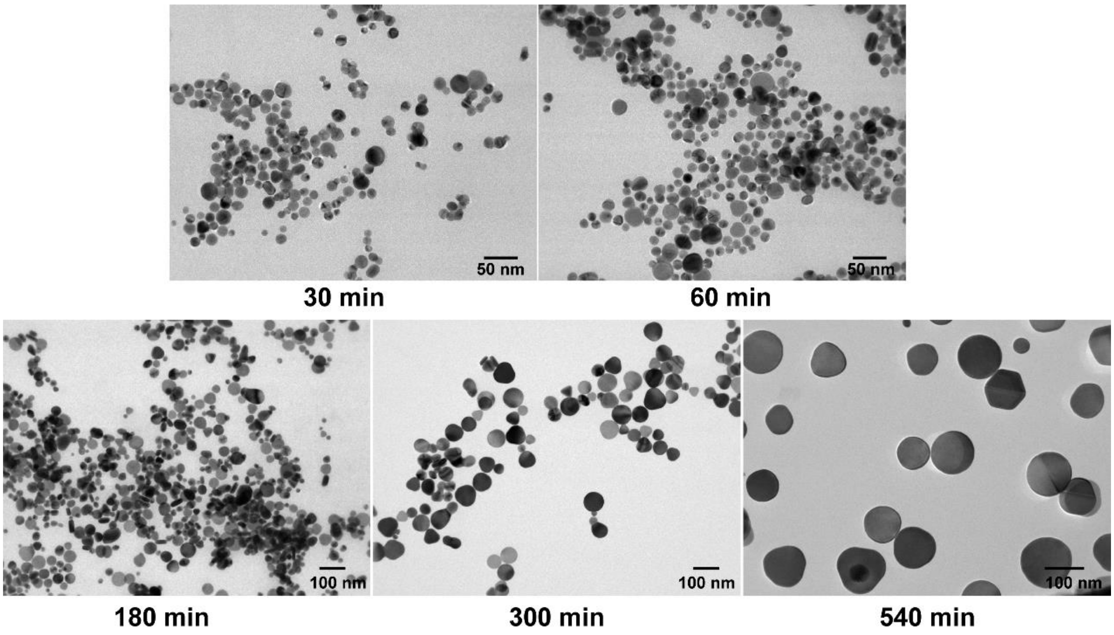

Supplementary Figure 9. TEM characterization of the conversion process of Au nanospheres to nanoplates. Time-dependent TEM images of Au nanostructures formed under irradiation of 500 ± 10 nm at 30, 60, 180, 300, and 540 min. The aggregation of different nanostructures can be found throughout the whole conversion process. Also, Au nanoplates were gradually generated with the increase of irradiation time. After 540 minutes of reaction, a high yield of Au nanoplates was formed accompanied by the disappearance of Au nanospheres.

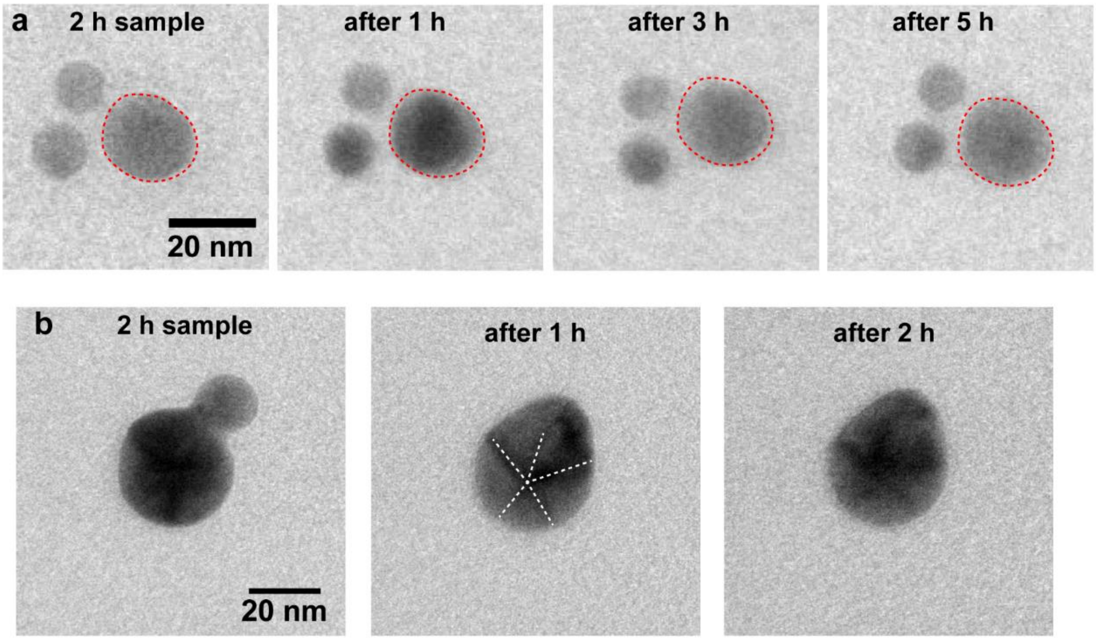

Supplementary Figure 10. TEM tracking of Au nanostructures evolution. TEM images of Au nanospheres (a) and nanoparticle-nanoparticle coalescence structures (b) from 2 h samples. No significant changes were found after 5 h of light-driven conversion in nanospheres of from 2h samples. The penta twinned structure does not exhibit significant etching and a smaller nanoparticle slowly integrates into it during the light irradiation.

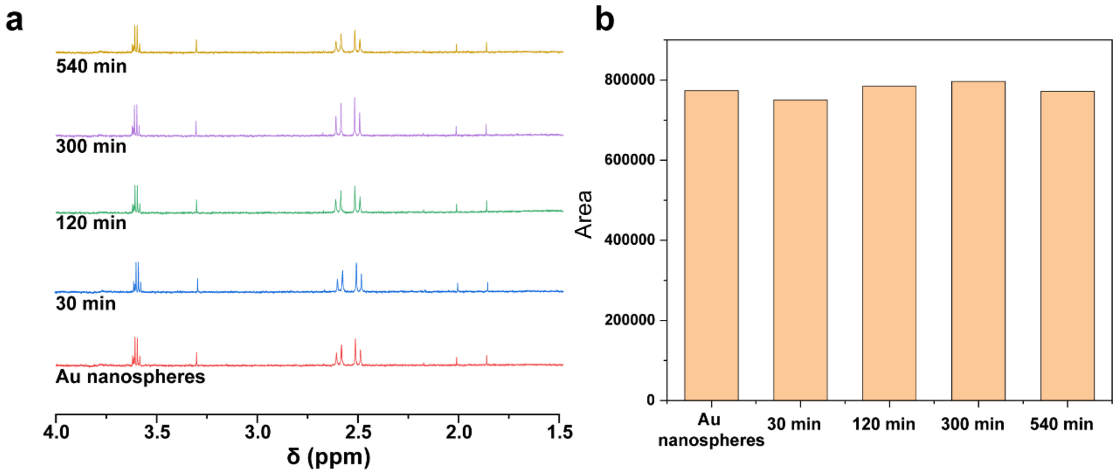

Supplementary Figure 11. 1H NMR spectroscopy tracking sodium citrate during the conversion process. (a) 1H NMR spectra for Au nanospheres solution and reaction solution after 30, 120, 300 and 540 min. (b) The corresponding integral area of the NMR peak of sodium citrate[6] (2.5 ppm) in **a.** Aliquots of sample (0.8 mL) were withdrawn and mixed with D_2O (0.2 mL) for NMR analysis. During the whole conversion, no new peaks appeared and there was no significant change in the integrated area of sodium citrate. These results suggest that oxidation and re-deposition are not the dominant mode in the whole morphological conversion process.

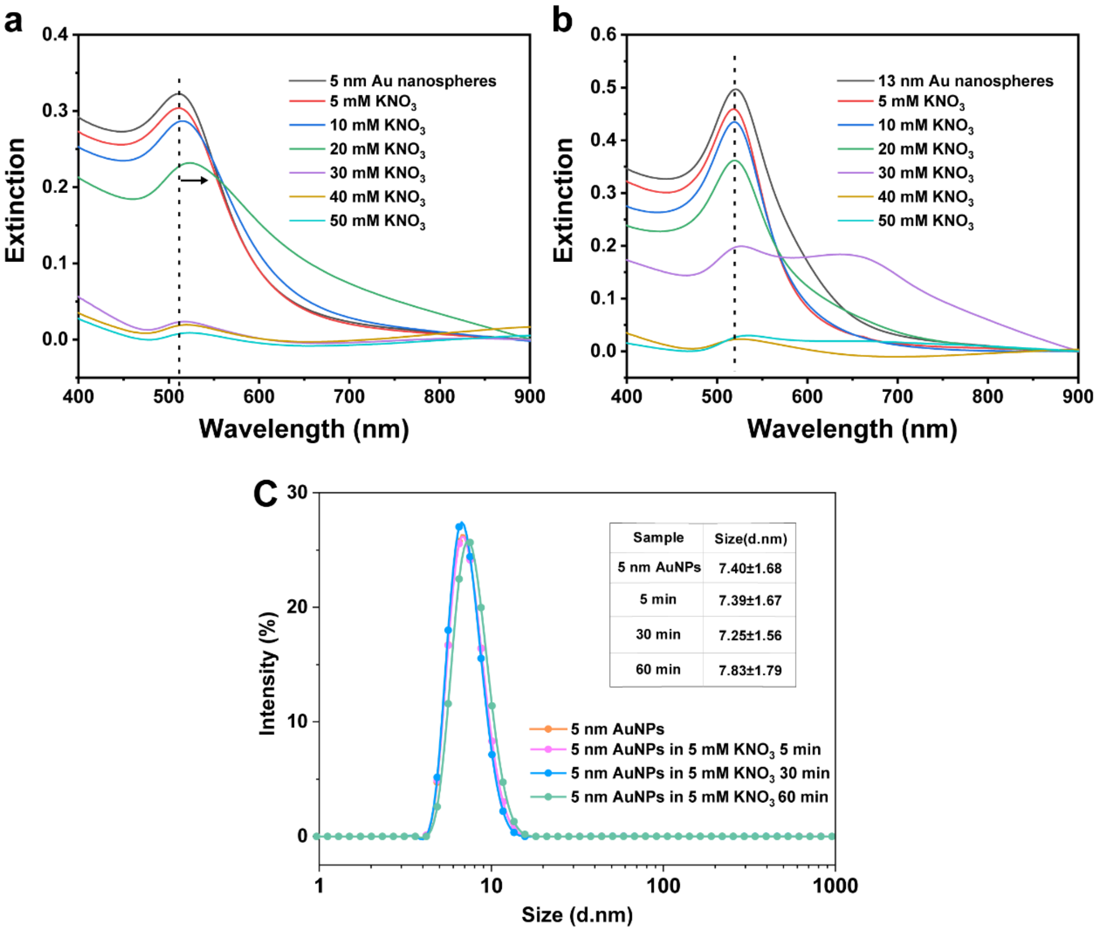

Supplementary Figure 12. Effect of salt concentration on Au nanoparticles. UV-Vis spectra of 5 nm Au nanospheres (a) and 13 nm Au nanospheres(b) at different salt concentrations. (c) Time-dependent DLS of 5 nm nanospheres in 5 mM KNO_3 solution. Since the high concentration of salt will reduce the thickness of counterion cloud (also known as the Debye screening length) on charged nanoparticles, which makes the van der Waal's attractive forces stronger than the repulsive electrostatic forces, allowing charged nanoparticles to aggregate. As confirmed by UV-Vis spectra (a), 5 nm nanospheres are unstable in solutions which concertation exceeds 10 mM KNO_3. 13 nm Au nanospheres (b) exhibited a shift of the SPR peak only at salt concentration above 30 mM KNO_3. These results indicate that larger nanoparticles are less susceptible to the salt concentration.

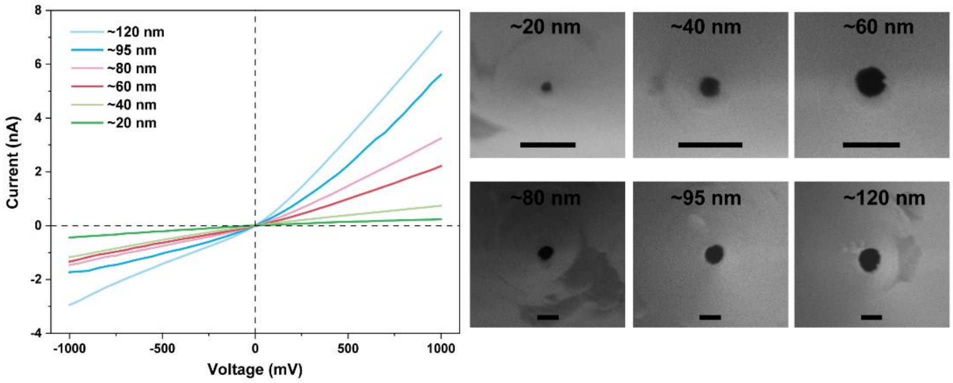

Supplementary Figure 13. Nanopore size characterization. Current-voltage curves and SEM images of nanopipettes with different nanopore sizes used in measurements. I-V curves obtained in 50 mM/5 mM trans/cis KNO_3 solution. All scale bars in all SEM images represent 100 nm. ~ 20 nm nanopores were employed for 5 nm Au nanospheres and 13 nm Au nanospheres translocation tests. ~ 40, ~ 60, ~ 95, and ~ 120 nm nanopores were used for 1, 3, 5, and 9 h sample translocation tests, respectively. ~ 80 nm nanopores were employed for 50 nm SiO_2 nanoparticles translocation tests.

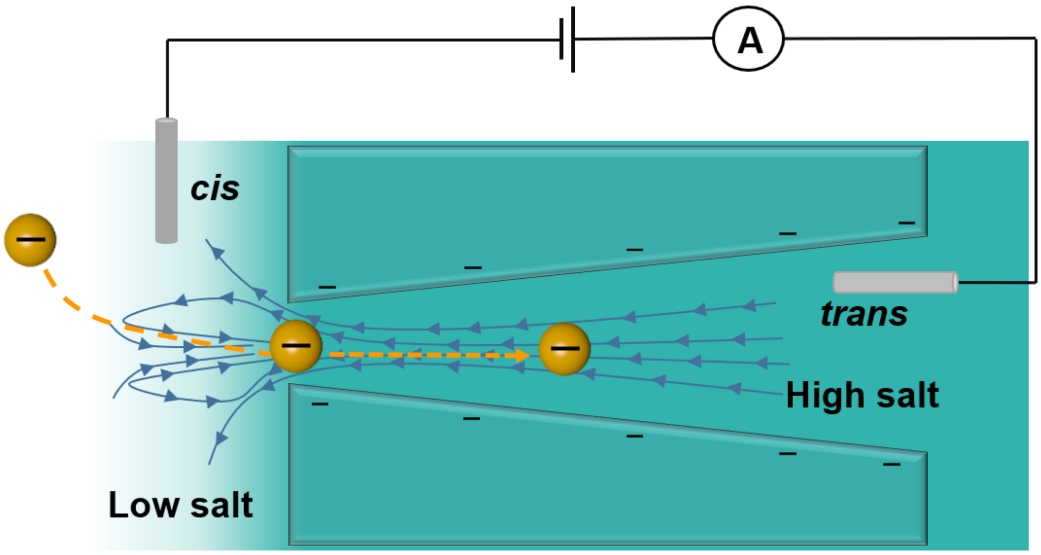

Supplementary Figure 14. Schematic illustration of a conical nanopore with a salt gradient for nanoparticles translocation. For a negatively charged conical nanopore, the salt concentration gradient creates an induced reverse electroosmotic flow (IREOF). This system has been theoretically verified in a previously reported work[7]. It provides the following two advantages in our experimental system: 1. IREOF improves the nanoparticle capture rate; 2. The low salt concentration on the *cis* side effectively prevents the aggregation of nanoparticles.

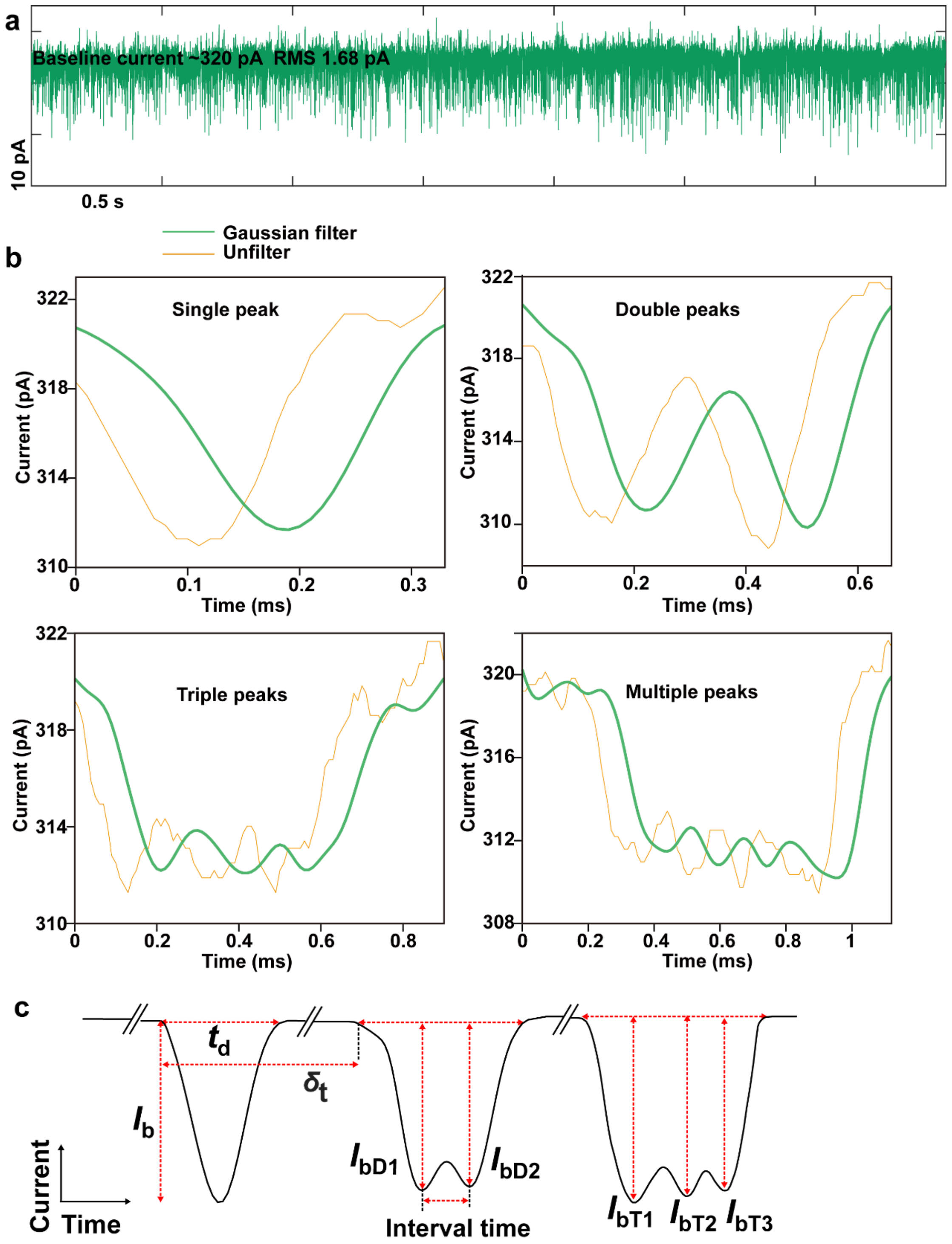

Supplementary Figure 15. Translocation study of 5 nm Au nanospheres. (a) Current versus time trace of Au nanospheres with stochastic current spikes. The data were obtained in 50 mM/5 mM trans/cis KNO_3. (b) Different current spikes of raw data from 5 nm Au nanospheres and after adding a 5 kHz Gaussian filter. (c) Spikes can be

characterized by current blockade (I_b), dwell time (t_d) and time between successive captured events (δ_t). The double peaks have two current blockades (I_{bD1} and I_{bD2}), and the time between the two blockades defines as the interval time. The triple peaks have three current blockades (I_{bT1} I_{bT2} and I_{bT3}), corresponding to two interval times.

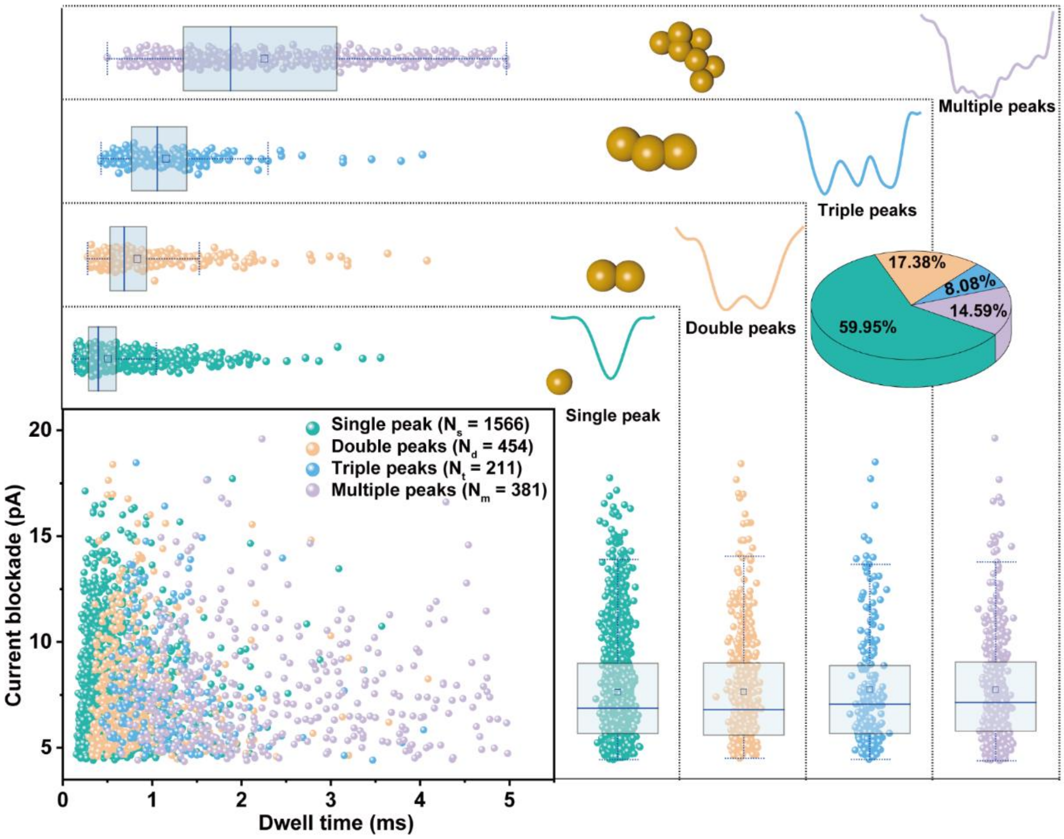

Supplementary Figure 16. Analysis and identification of translocation events of initial 5 nm Au nanospheres. Scatter plots of t_d vs. I_b combined with the box plots. Box plots of I_b (right) distribution show that the means, medians, quartiles and ranges of single-peak, double-peak, triple-peak and multiple-peak events are close. And Box plots of t_d (top) distribution demonstrate apparent differences in the mean, median, quartiles and range of the four types of peaks. The pie chart shows the relative proportion of four types of peaks in 5 nm Au nanospheres**.** Single peak (N_s = 1566): 59.95%, double peaks (N_d = 454): 17.38%, triple peaks (N_t = 211): 8.08% and multiple peaks (Nm = 381): 14.59%. Box plots show median (blue line), mean (blue square box), quartiles (boxes) and range (whiskers).

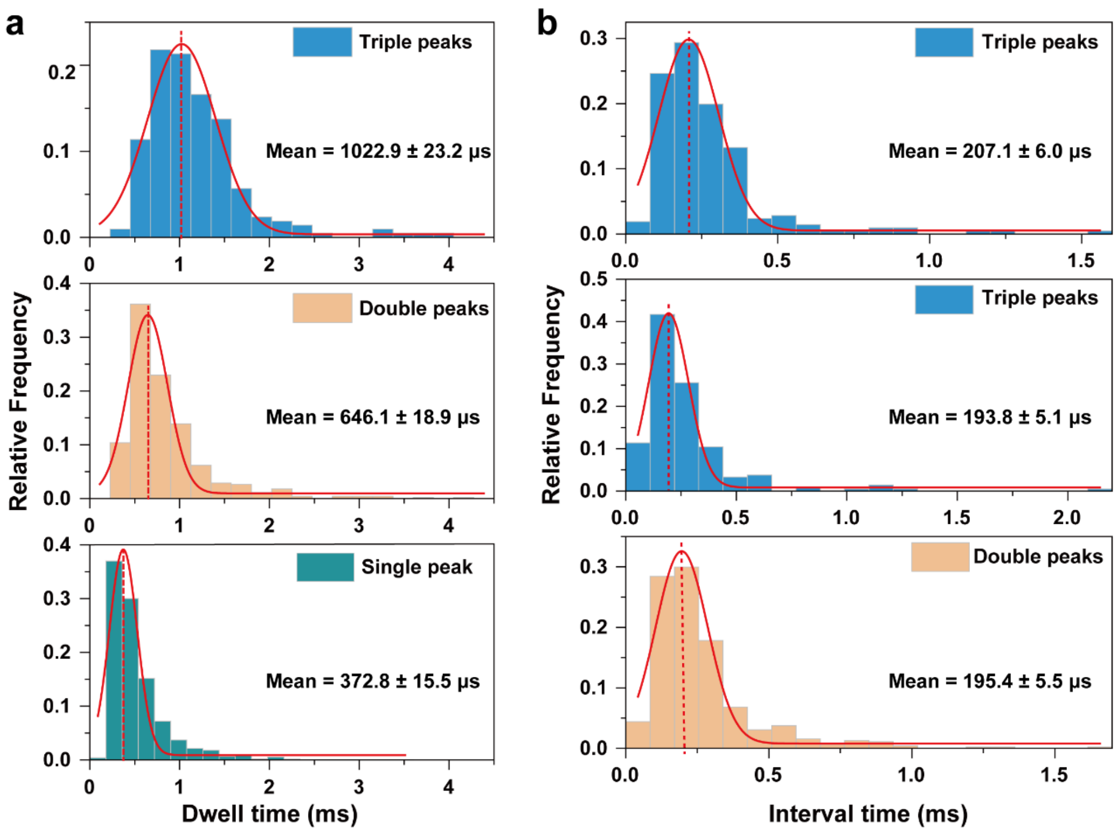

Supplementary Figure 17. Analysis translocation events of 5 nm Au nanospheres. Histograms of the dwell time (a) and interval time (b). Gaussian fits to the dwell time of the single peak, double peaks and triple peaks produced peaks centered at 372.8 μs, 646.1 μs and 1022.9 μs, respectively. The interval time distribution peaked at 195.4 μs for the double peaks and 193.8 μs and 207.1 μs for the triple peaks.

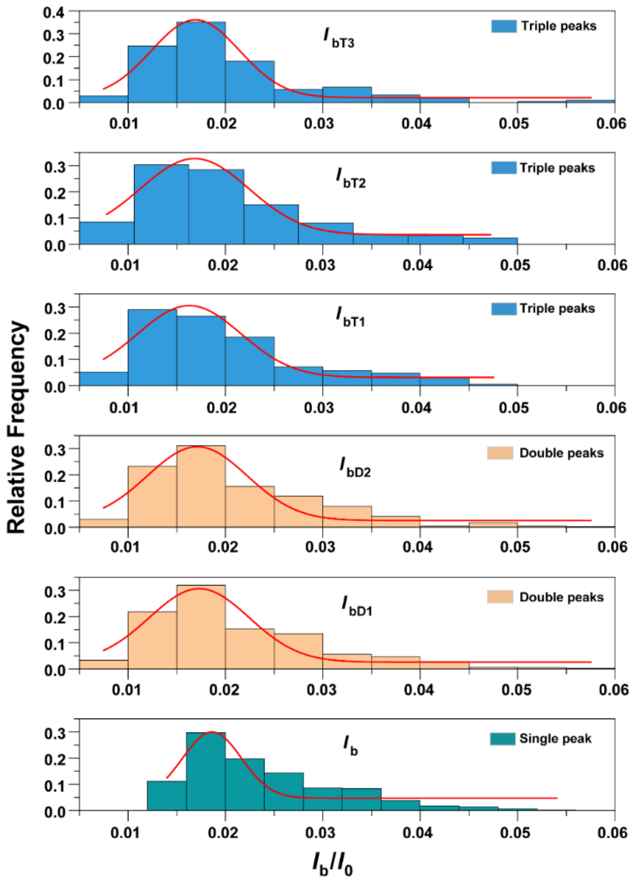

Supplementary Figure 18. Analysis translocation events of 5 nm Au nanospheres.

Histograms of I_b/I_0 translocation events of Au nanospheres**.** The statistical mean value of I_b/I_0 for each of these peaks was very close.

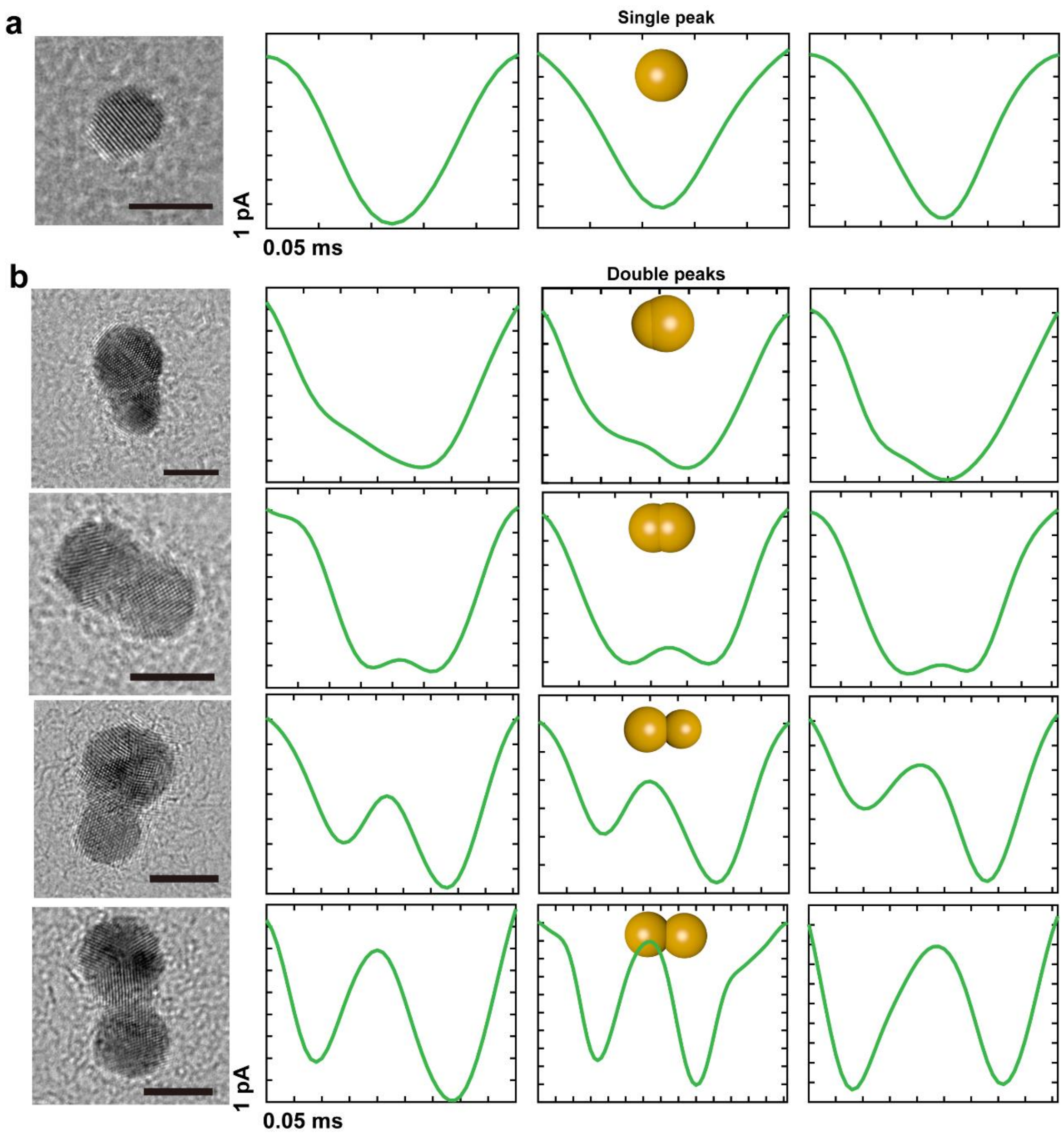

Supplementary Figure 19. Typical nanopore current–time peaks shape and possible structures with TEM images from Au nanospheres. (a) Single peak events and TEM image. (b) The double peaks event and TEM images**.** All scale bars represent 5 nm.

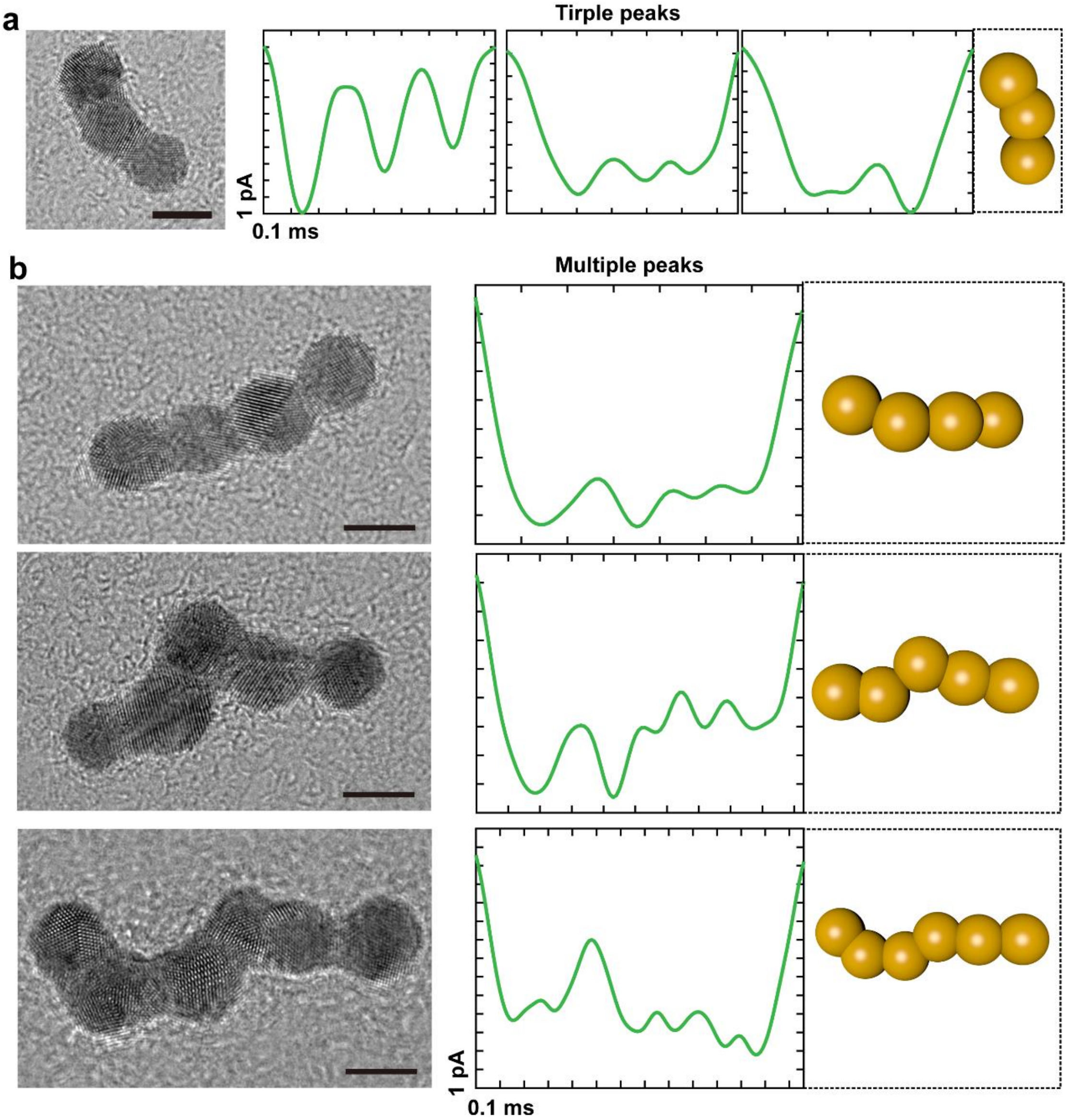

Supplementary Figure 20. Typical nanopore current–time peaks shape and possible structures with TEM images from Au nanospheres. Triple peaks (a) and multiple peaks (b) were also observed in translocation events, and TEM images showed three Au nanospheres and multiple Au nanospheres in different aggregated states. All scale bars represent 5 nm.

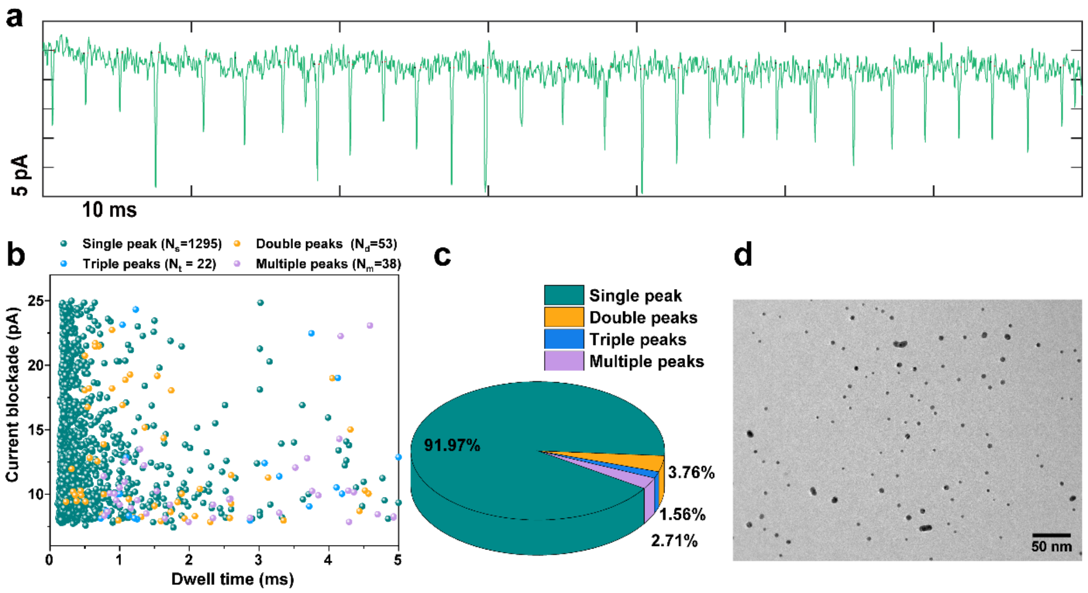

Supplementary Figure 21. Analysis translocation events of Au nanospheres (after centrifugation). (a) Concatenate events from Au nanospheres (7040 × g, 15 min) translocation test using a ~20 nm quartz nanopore at a 1 V bias voltage. (b) Scatter plots of t_d vs. $I_{b.}$ (c) Pie chart of four types of peaks in a total of 1408 translocation events. The percentage of single, double, triple, and multiple peaks were 91.97%, 3.76%, 1.56% and 2.71%, respectively. (d) TEM image of 5 nm Au nanospheres from supernatant after centrifugation.

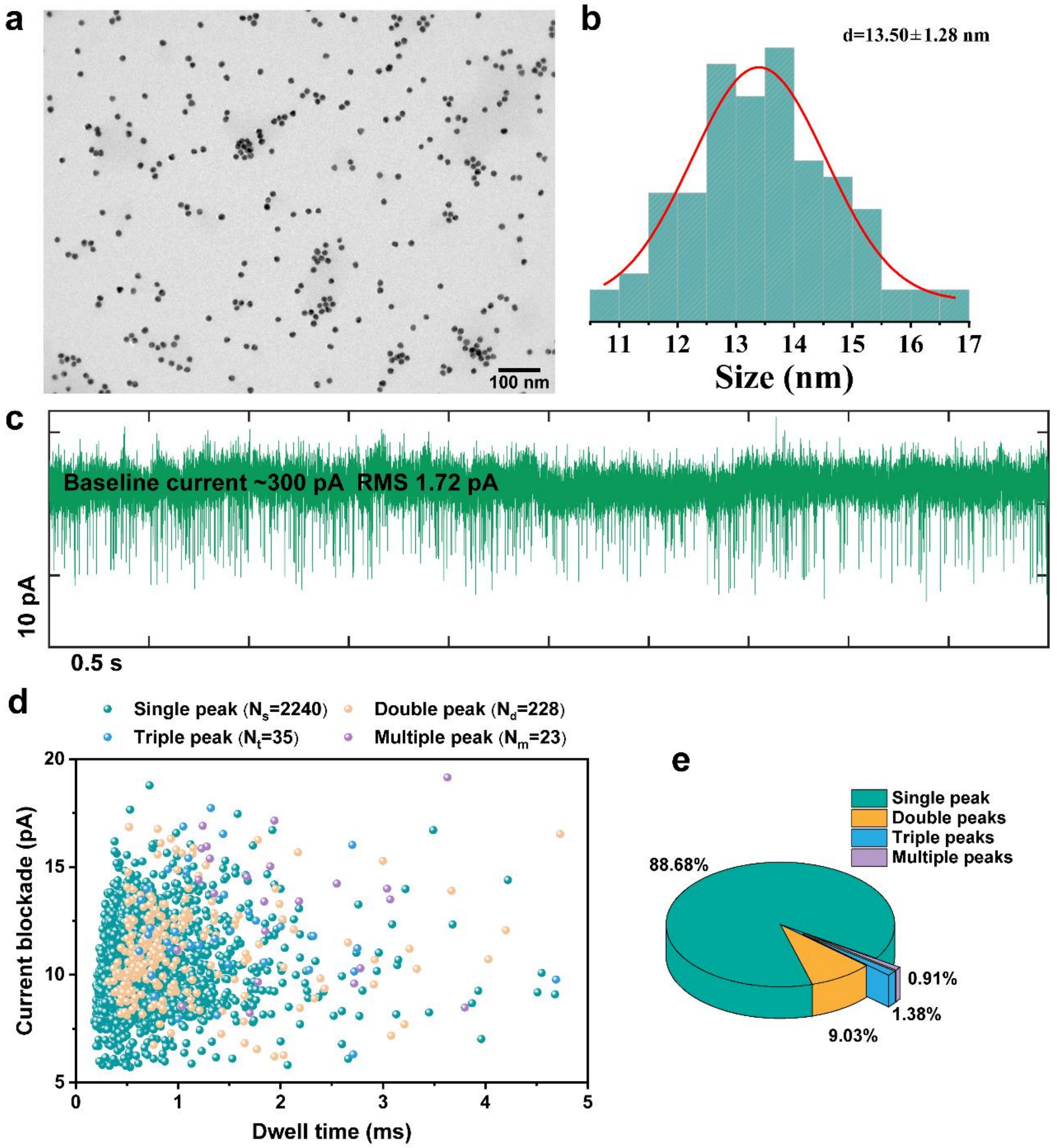

Supplementary Figure 22. Analysis translocation events of 13 nm Au nanospheres. (a) TEM image of 13 nm Au nanospheres shows much better monodispersity compared to 5 nm Au nanospheres in Supplementary Figure 1b. (b) Histogram of Au nanospheres size exhibits a Gaussian distribution with an average diameter of d = 13.50 ± 1.28 nm. (c) Current versus time trace of 13 nm Au nanospheres. (d) Scatter plots of t_d vs. $I_{b.}$ (e) Pie chart of four types of peaks in 13 nm translocation events. A total of 2526 events, the

percentage of single, double, triple, and multiple peaks were 88.68%, 9.03%, 1.38% and 0.91%, respectively. These results suggest that the salt concentration gradient (50 mM/5 mM trans/cis KNO_3 solution) approach is a good strategy for analyzing nanoparticle and avoiding additional aggregation. The low salt concentration at the *cis* end contributes to the maintenance of Debye screening length on the surface of the nanoparticles.

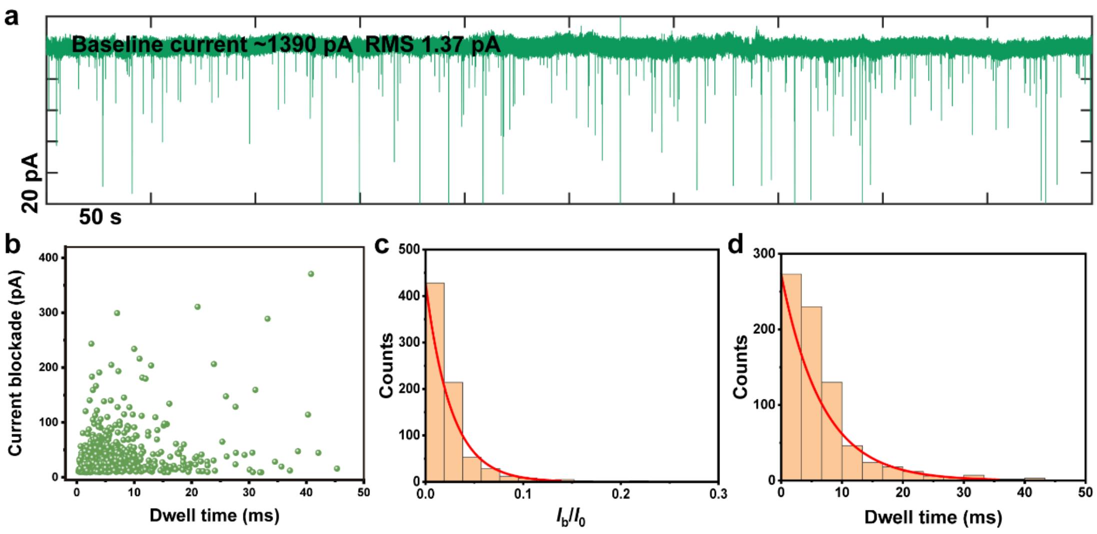

Supplementary Figure 23. Translocation study of Au nanoplates. (a) Current versus time trace of Au nanoplates. The data were obtained in 50 mM/5 mM *trans*/*cis* KNO_3 using a ~120 nm nanopore at a bias voltage of 300 mV. (b) Scatter plots of t_d vs. I_b. (c-d) Histogram of I_b/I_0 and t_d.

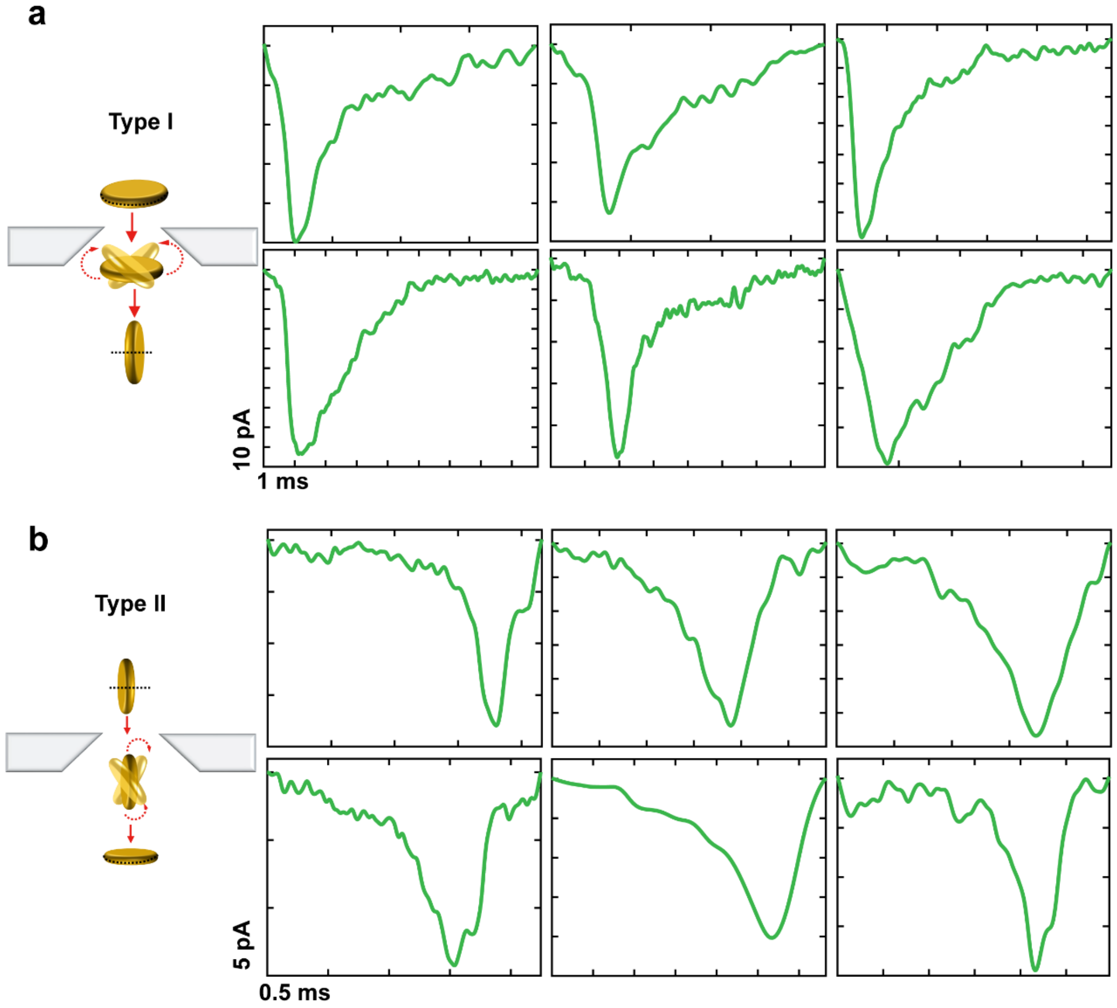

Supplementary Figure 24. Nanopore analysis of Au nanoplates. Type I (a) and type II (b) of current–time peak shape for Au nanoplates.

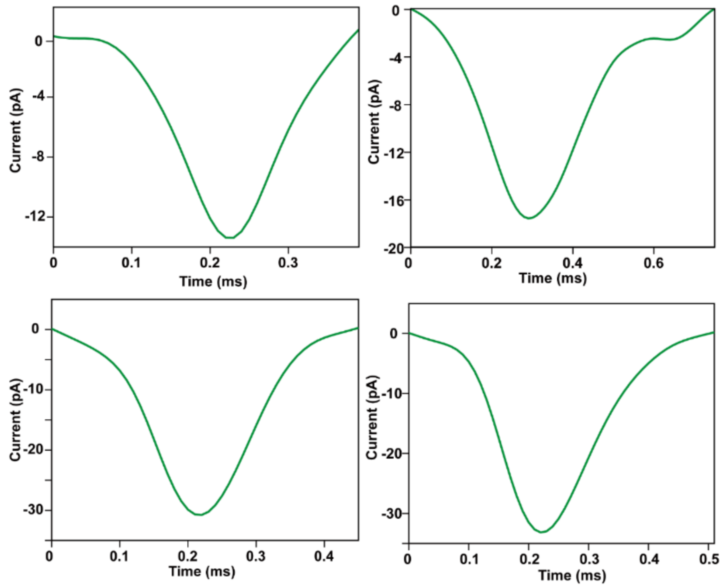

Supplementary Figure 25. Nanopore analysis of byproduct Au nanospheres in Au nanoplates sample. Au nanospheres events in Au nanoplates sample.

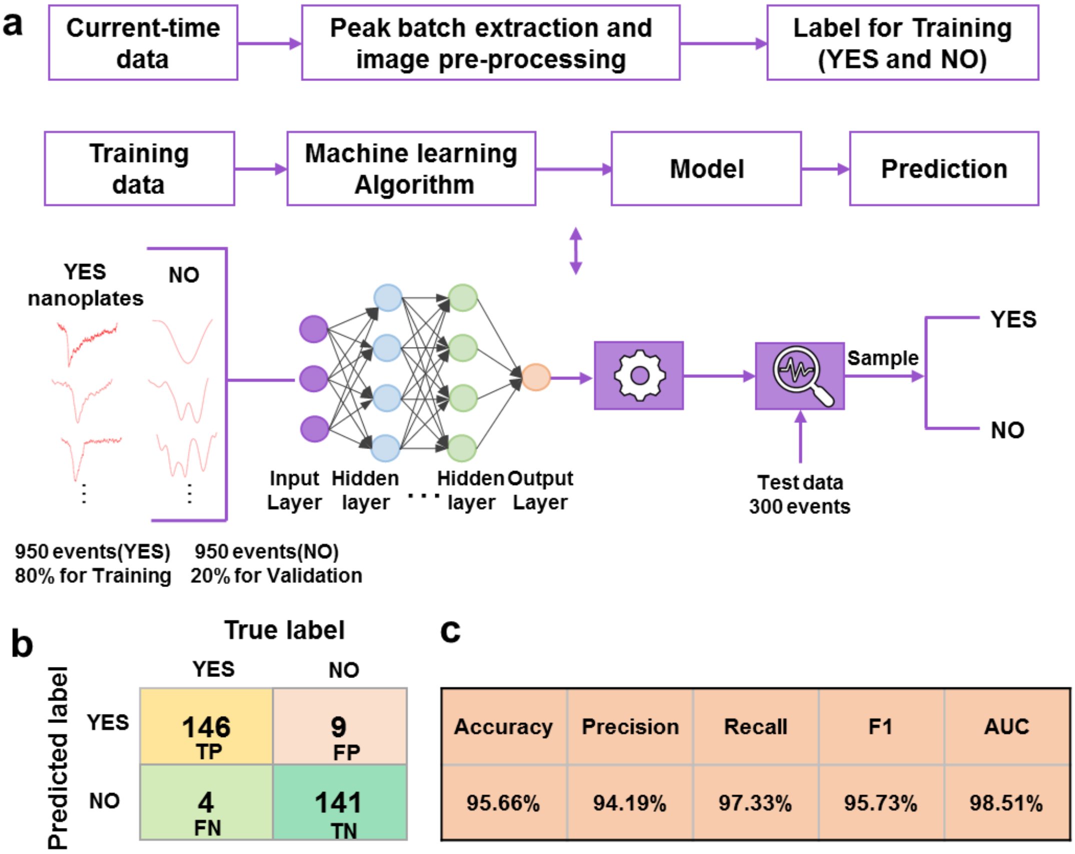

Supplementary Figure 26. Identification events of nanoplates using supervised machine learning. (a) Learning process of Au nanoplates events using CNN learning algorithm. Taking into account the differences in the event peak shapes of Au nanoplates and Au nanospheres, and their overlap in I_b, we developed peak shape based machine learning to classify them. All events are extracted from nanoplates and nanospheres. Out of the 2200 images without any axis information (1100 images identified as nanoplates are labeled YES and the 1100 non-nanoplates images are labeled NO.) provided in the dataset, 1900 are given for training and 300 are given for testing. The CNN model was implemented in Python 3.8 using Keras (v1.1.2) from Tensorflow (v2.3.0). (b) Confusion matrix of test data using model prediction. The numbers in the matrix cells indicate the number of events.

TP and TN represent true positive and true negative, respectively. FP and FN indicate false positive and false negative, respectively.

The model was evaluated by accuracy [(TP+TN)/(TP+TN+FP+FN)], precision [TP/(TP+FP)], recall [TP/(TP+FN)], F1 measure [(2×precision×recall)/(precision+recall)], and an area under the curve (AUC). As shown in (c), we have gotten to 95.66% of accuracy on test dataset.

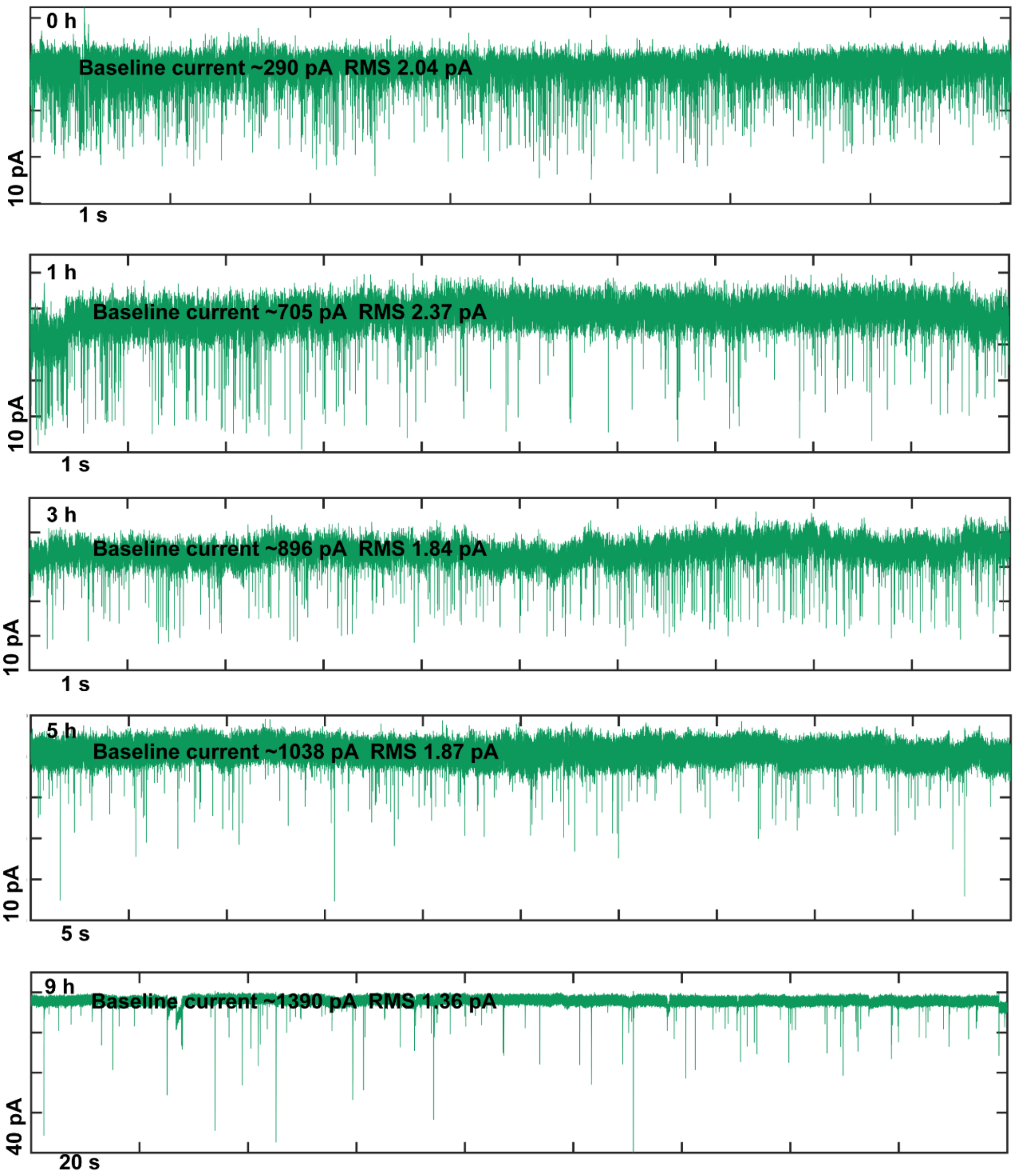

Supplementary Figure 27. Nanopore analysis of samples from different conversion time. Current versus time trace of the sample solution at 0 h 1 h, 3 h, 5 h and 9 h during the reaction. ~ 20, ~ 40, ~ 60, ~ 95, and ~ 120 nm nanopores were used for 0 h, 1 h, 3 h, 5 h, and 9 h sample translocation tests, respectively. The applied bias voltages were 1 V, 1 V, 500 mV, 400 mV and 300 mV for the 0 h, 1 h, 3 h, 5 h and 9 h measurements, respectively.

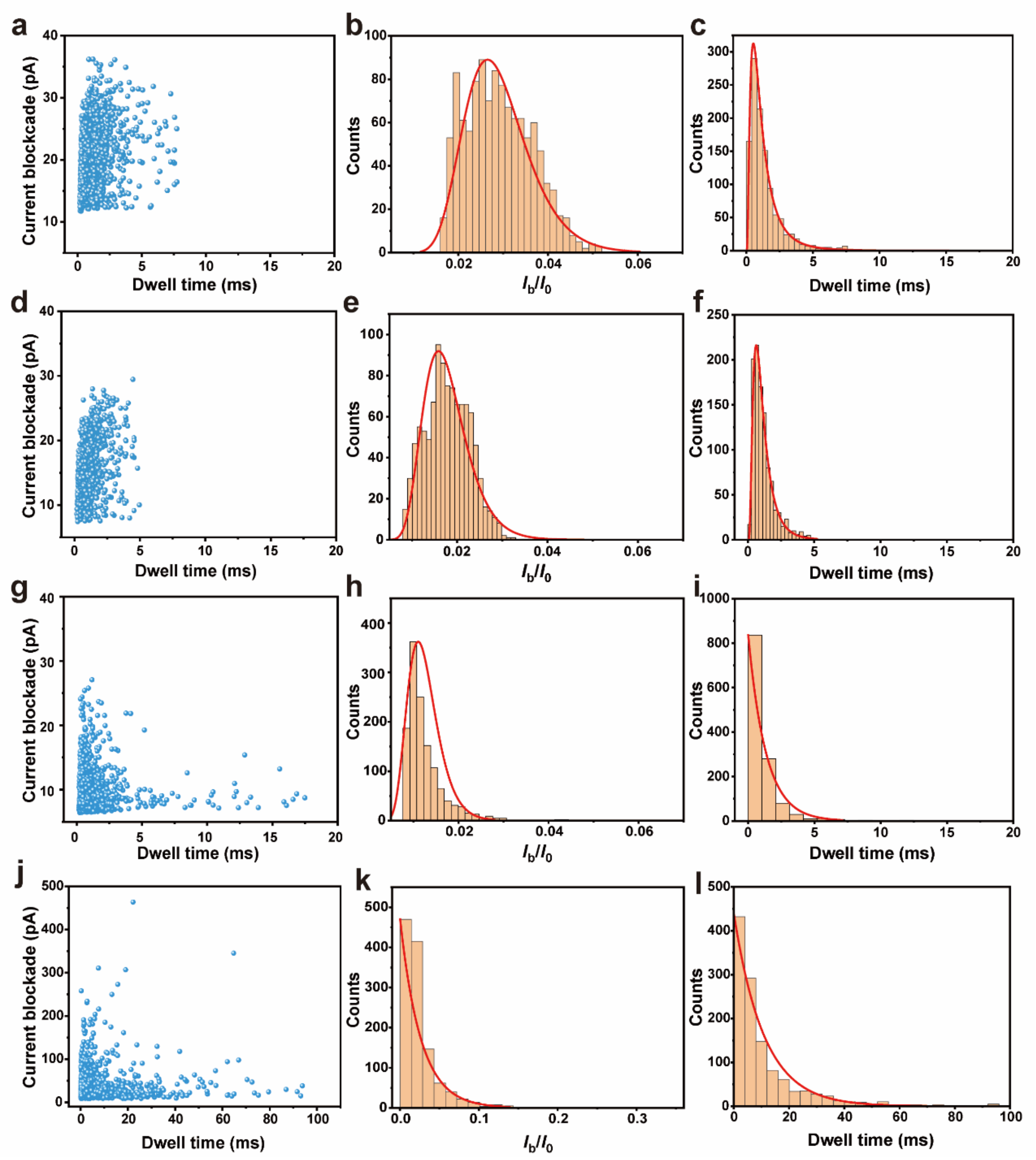

Supplementary Figure 28. Nanopore analysis of samples from the conversion process. Scatter plots of t_d vs. I_b obtained from 1 h (a), 3 h (d), 5 h (g) and 9 h (j). Histogram of I_b/I_0 obtained from 1 h (b), 3 h (e), 5 h (h) and 9 h (k). Histogram of t_d obtained from 1 h (c), 3 h (f), 5 h (i) and 9 h (l). 1135, 1035, 1283, 1198 events were obtained from 1 h, 3 h, 5 h, 9 h samples.

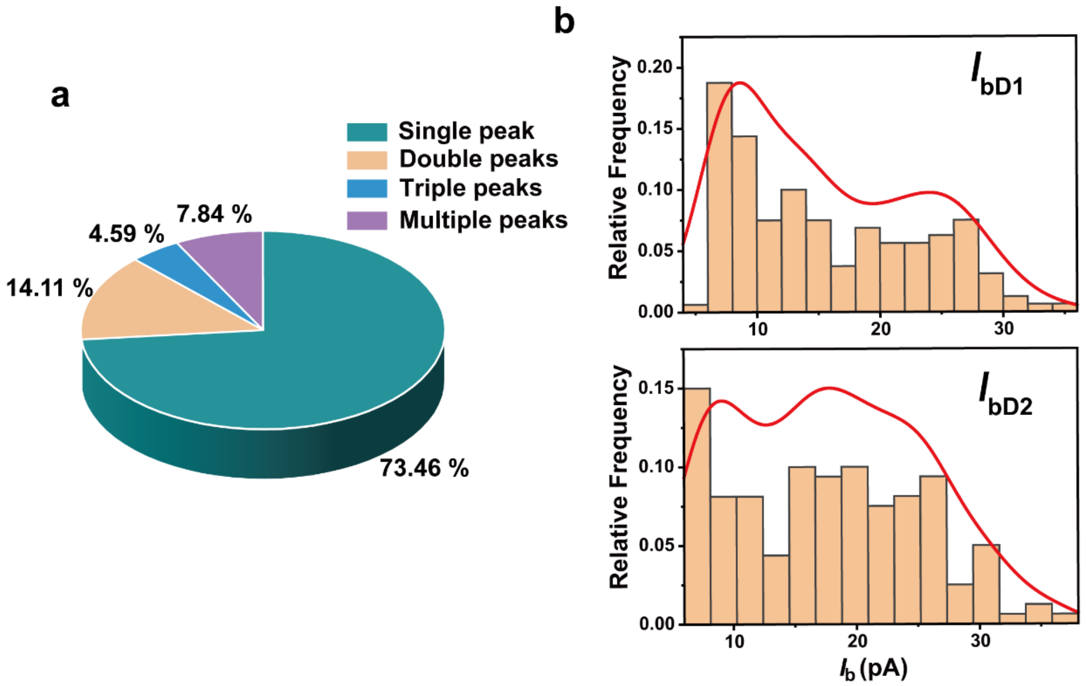

Supplementary Figure 29. Analysis translocation events of 1 h sample. (a) Pie chart of four types of peaks in 1 h sample translocation events. A total of 1134 events, the percentage of single, double, triple, and multiple peaks were 73.46%, 14.11%, 4.59% and 7.84%, respectively. (b) Histogram of double peaks (I_{bD1} and I_{bD2}). Kernel smooth curve shows two probability distributions.

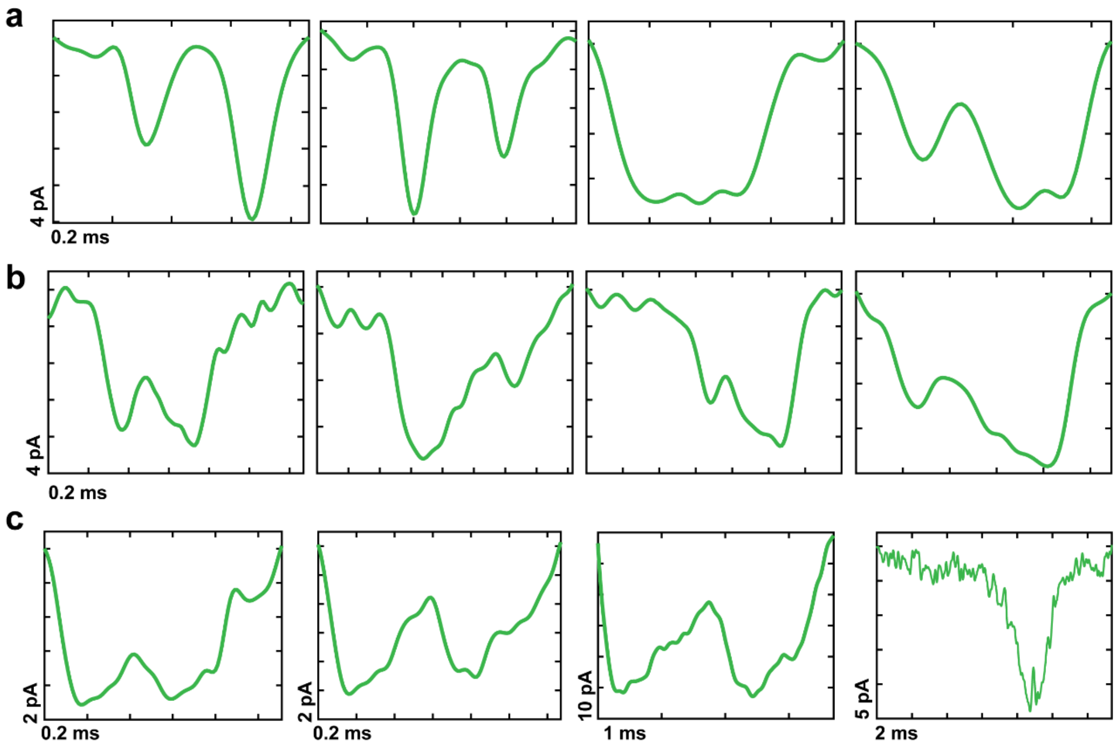

Supplementary Figure 30. Nanopore analysis of intermediate coalescence species.

Typical nanopore current–time peak shapes for nanoparticle-nanoparticle (a), nanoparticle-nanoplate (b) and nanoplate-nanoplate coalescence (c).

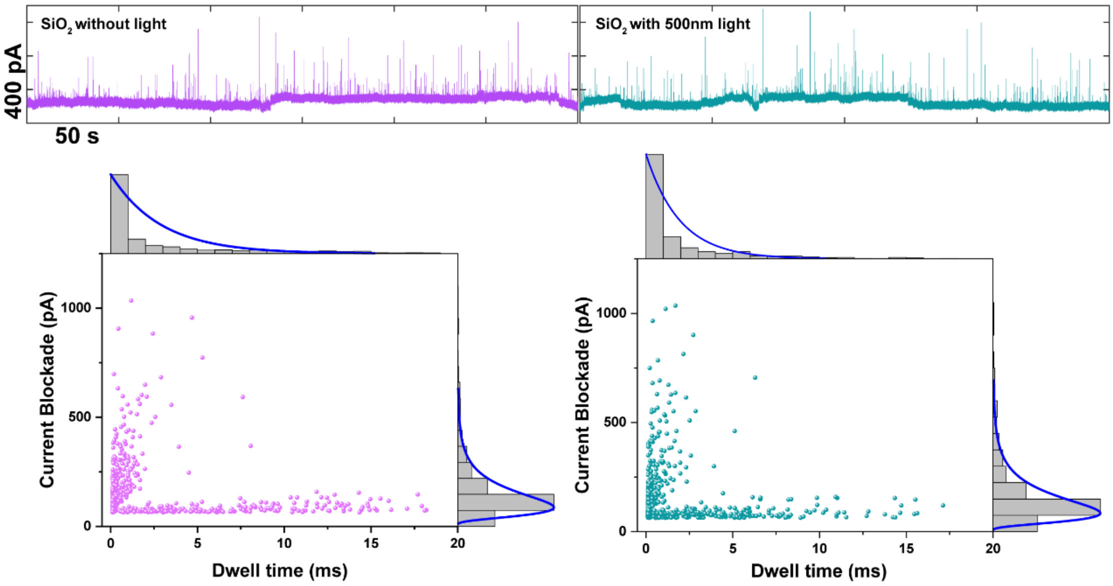

Supplementary Figure 31. Nanopore analysis of the function of light. Current versus time trace of SiO_2 nanospheres without light and with light (up), and corresponding scatter plots of t_d vs. I_b (down). Measurements were collected using a ~80 nm nanopore at a bias voltage of -1 V. The baseline current is about -2250 pA and RMS is 12.73 pA.

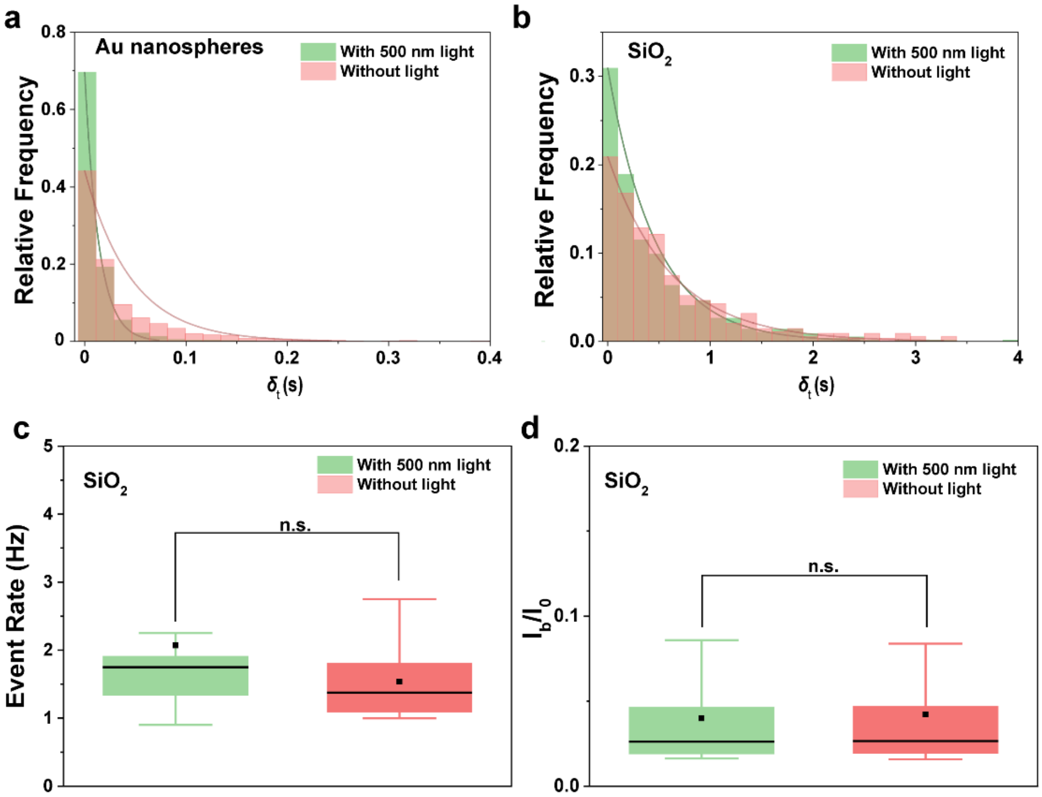

Supplementary Figure 32. Nanopore analysis of the role of light on morphology evolution. (a) Histogram of δ_t Au nanospheres without light and with light. (b) Histogram of δ_t SiO_2 nanospheres without light and with light. (c) Box plots of event rate SiO_2 nanospheres with 500 nm light and without light. Mann–Whitney *U* tests, $p = 0.136 > 0.05$. n.s., not significant. (d) Box plots of I_b/I_0 from SiO_2 nanospheres with 500 nm light and without light. Mann–Whitney *U* tests, $p = 0.800 > 0.05$. n.s., not significant. Box plots show median (black line), mean (black square box), quartiles (boxes) and range (whiskers).

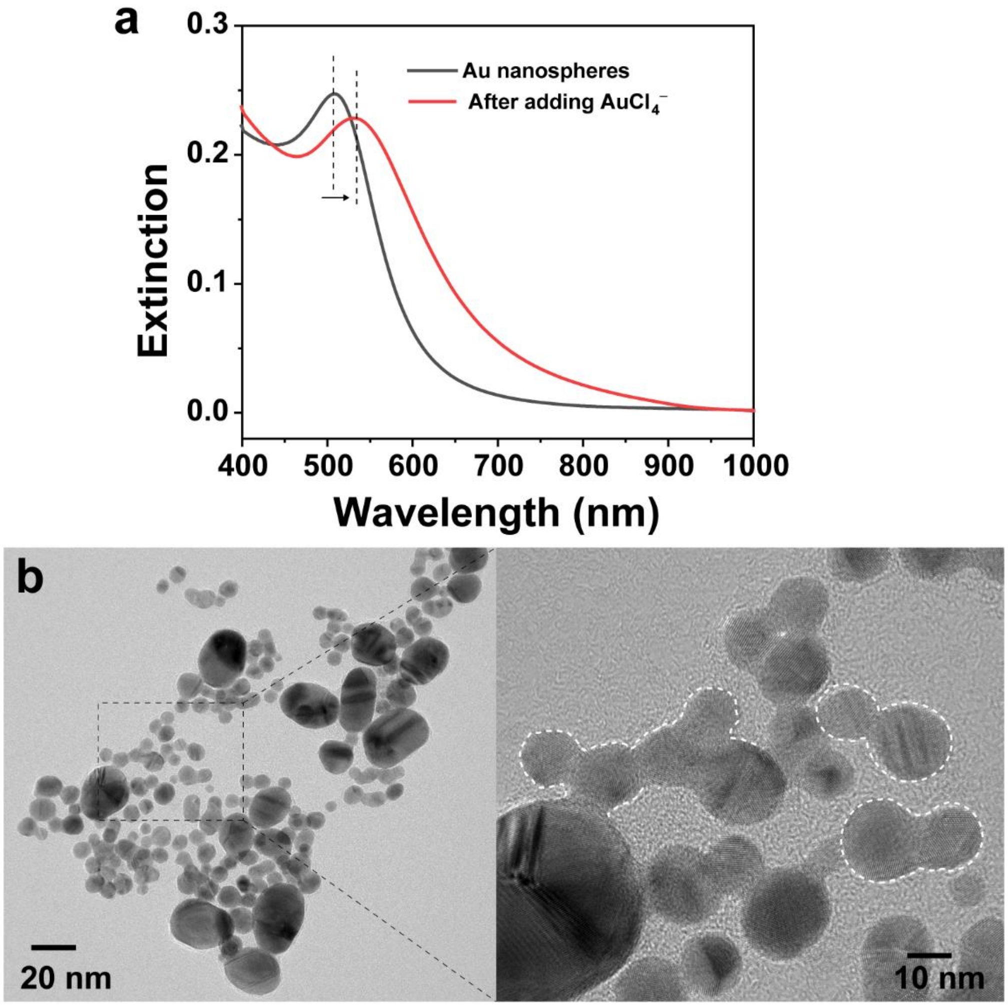

Supplementary Figure 33. Effect of high concentration of $HAuCl_4$ on the conversion of Au nanospheres to aggregates. (a) UV−vis spectra of Au nanospheres and after adding 100 μl of 10 mM $HAuCl_4$. (b) TEM images of gold nanostructures from the reaction solution by illumination (500 ± 10 nm) for 540 min. After adding the $AuCl_4^-$, the peak of Au nanospheres immediately decreased in intensity accompanied by red-shift. The Au nanospheres were aggregated together and grow with irregular shapes by illumination for 540 min. The results show that the $AuCl_4^-$ can oxidize Au^0 and remove the surfactant (trisodium citrate) to induce the aggregation of Au nanospheres.

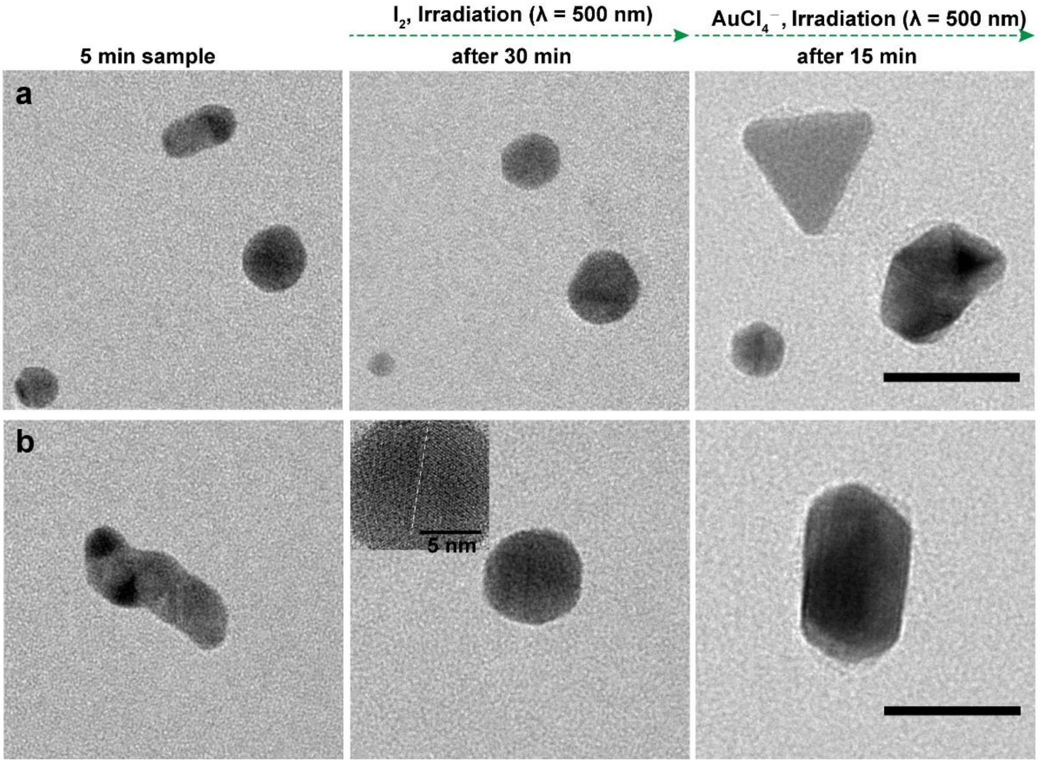

Supplementary Figure 34. TEM images of the tracking of nanoparticle aggregates conversion process. The first reaction is to put the TEM chip in the real conversion solution for 30 min with 500 nm light irradiation (the second row). The second reaction is to further put the TEM chip in a solution containing 4.84 ml ultrapure H_2O, 0.50 ml PVP (K30, 5 mg/ml), 0.50 ml CH_3OH, and 0.16 ml $HAuCl_4$ (25 mM) for 15 min under 500 nm light irradiation (the third row). All scale bars indicate 20 nm.

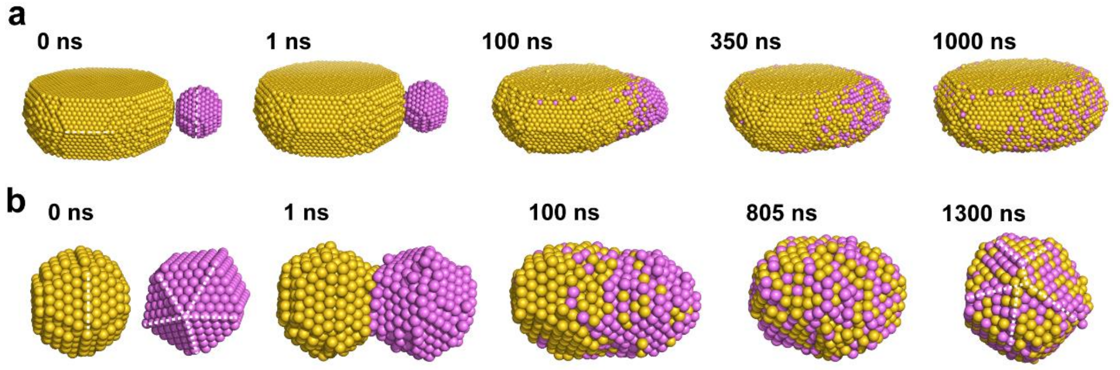

Supplementary Figure 35. MD simulations of nanoparticles coalescence evolution process. (a) Snapshots of the coalescence trajectory of a larger Au nanoplate (gold) and planar twinned structure (pink). Dashed white lines serve as a guide to highlight the twin boundary. (b) Snapshots of the coalescence trajectory of two Au nanospheres with similar size, planar twinned (gold) and penta twinned (pink) structure.

Supplementary Table 1. The detailed parameters for the preparation of nanopores with different diameters.

Heat	Filament	Velocity	Delay	Pull	Tip size (nm)	Capillary
700	4	60	145	175	~ 20	QF100-70-7.5
750	3	20	128	140	~ 40	QF100-50-7.5
700	3	20	128	140	~ 60	QF100-50-7.5
680	3	20	128	140	~ 80	QF100-50-7.5
695	3	20	140	150	~ 95	QF100-50-7.5
700	3	18	128	140	~ 120	QF100-50-7.5

Supplementary References

1. Greenwood, N. N. & Earnshaw, A. *Chemistry of the Elements*. 853-859 (Elsevier, 2012).
2. Pursell, J. L. & Pursell, C. J. Host–guest inclusion complexation of α-cyclodextrin and triiodide examined using UV–Vis spectrophotometry. *J. Phys. Chem. A* **120**, 2144-2149 (2016).
3. Bhagat, P. *et al.* Molecular iodine preconcentration and determination in aqueous samples using poly (vinylpyrrolidone) containing membranes. *Talanta* **74**, 1313-1320 (2008).
4. Stangherlin, S., Cathcart, N., Sato, F. & Kitaev, V. Gold nanoprisms: synthetic approaches for mastering plasmonic properties and implications for biomedical applications. *ACS Appl. Nano Mater.* **3**, 8304-8318 (2020).
5. Chen, L. *et al.* High-yield seedless synthesis of triangular gold nanoplates through oxidative etching. *Nano lett.* **14**, 7201-7206 (2014).
6. Xue, C., Metraux, G. S., Millstone, J. E. & Mirkin, C. A. Mechanistic study of photomediated triangular silver nanoprism growth. *J. Am. Chem. Soc.* **130**, 8337-8344 (2008).
7. Hsu, W. L. & Daiguji, H. Manipulation of protein translocation through nanopores by flow field control and application to nanopore sensors. *Anal. Chem.* **88**, 9251-9258 (2016).
