# Supplementary material for: Identification of plasmon-driven nanoparticle-coalescence-dominated growth of gold nanoplates through nanopore sensing
Source: Nat Commun. 2022 Mar 17;13:1402. doi: 10.1038/s41467-022-29123-9 (PMC8931024; doi:10.1038/s41467-022-29123-9)
Supplement: Supplementary file 3 — Description of Additional Supplementary [file 41467_2022_29123_MOESM3_ESM.doc]

**Description of Additional Supplementary Files**

**Supplementary Video 1**

Electron beam induces the fusion of I_2_-treated Au nanoparticles.

**Supplementary Video 2**

Molecular dynamics simulation shows the coalescence process of a larger Au nanoplate (gold) and penta twinned structure (pink).

**Supplementary Video 3**

Molecular dynamics simulation shows the coalescence process of a larger Au nanoplate (gold) and planar twinned structure (pink).

**Supplementary Video 4**

Molecular dynamics simulation shows the coalescence process of two Au nanospheres with similar size, planar twinned (gold) and penta twinned (pink) structure.
